# Supplementary material for: Mechanism and mitigation of stainless steel dissolution in LiFSI-based lithium-ion battery electrolytes
Source: Nat Commun. 2026 Apr 28;17:3866. doi: 10.1038/s41467-026-72530-5 (PMC13125316; doi:10.1038/s41467-026-72530-5)
Supplement: Supplementary file 1 — Supplementary Information [file 41467_2026_72530_MOESM1_ESM.pdf]

Supplementary Information

Mechanism and Mitigation of Stainless Steel Dissolution in LiFSI based Lithium Ion Battery Electrolytes

Peng Yan[1], Marian Cristian Stan[1], Kazem Zhour[2,3], Diddo Diddens[1], Christian Wölke[1], Rayan Guerdelli[1], Martin Winter[1,4] and Isidora Cekic-Laskovic[1]*

[1]Helmholtz-Institute Münster (HI MS), IMD-4, Forschungszentrum Jülich GmbH, Corrensstraße 48, 48149 Münster, Germany

[2]Institute of Physical Chemistry, University of Münster, Corrensstraße 28, 48149 Münster, Germany

[3]Multiscale Modelling of Heterogeneous Catalysis in Energy Systems, RWTH Aachen University, 52062 Aachen, Germany

[4]MEET Battery Research Center, University of Münster, Corrensstraße 46, 48149 Münster, Germany

Supplementary Figures

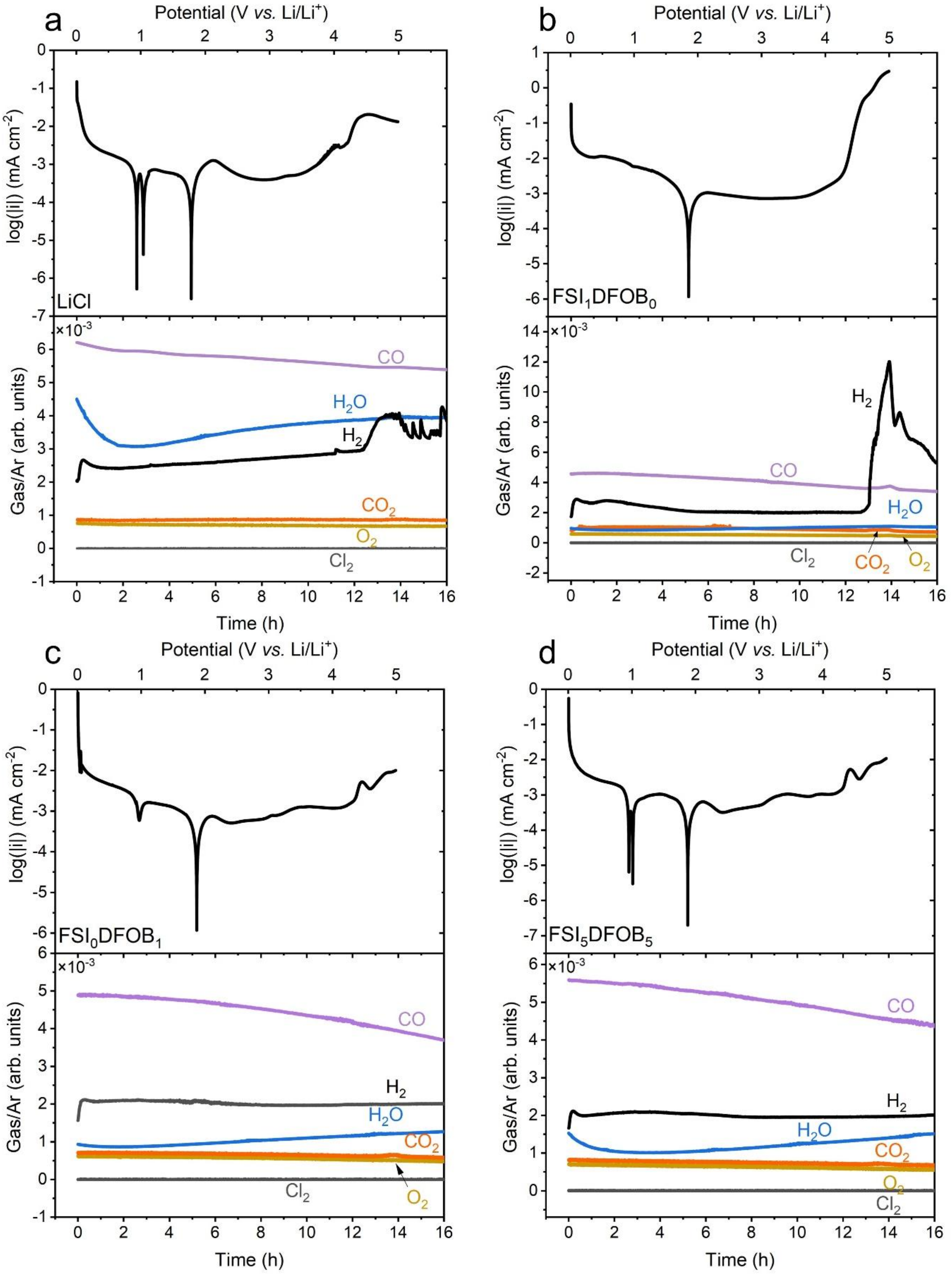

Figure S 1. DEMS analysis of gas evolution on SUS316 electrodes. Linear sweep voltammograms and corresponding mass spectrometry signals for 3-electrode DEMS cells scanned using SUS316 as a working electrode in (a) LiCl, (b) FSI_1DFOB_0, (c) FSI_0DFOB_1, (d) FSI_5DFOB_5 electrolytes. LSV curves were recorded at a scan rate of 0.1 mV s^{-1} from 0 to 5 V *vs.* Li/Li^+ at 25 °C. The following gases were measured: H_2 (m/z=2), CO_2 (m/z=44), CO (m/z=28), O_2 (m/z=32), H_2O (m/z=18), Cl_2 (m/z=70, 72, 74) and all gases were normalized to the Ar signal to eliminate the fluctuation of the carrier gas.

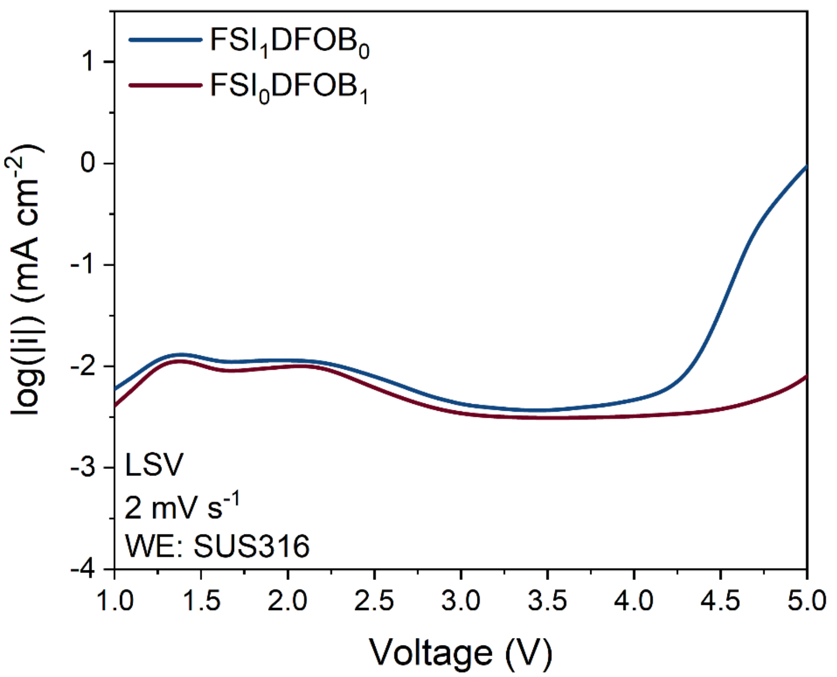

Figure S 2. Electrochemical stability of SUS316 in LiFSI and LiDFOB-based electrolytes. Linear sweep voltammogram of cells containing SUS316 as working electrode with FSI_1DFOB_0 or FSI_0DFOB_1 electrolyte.

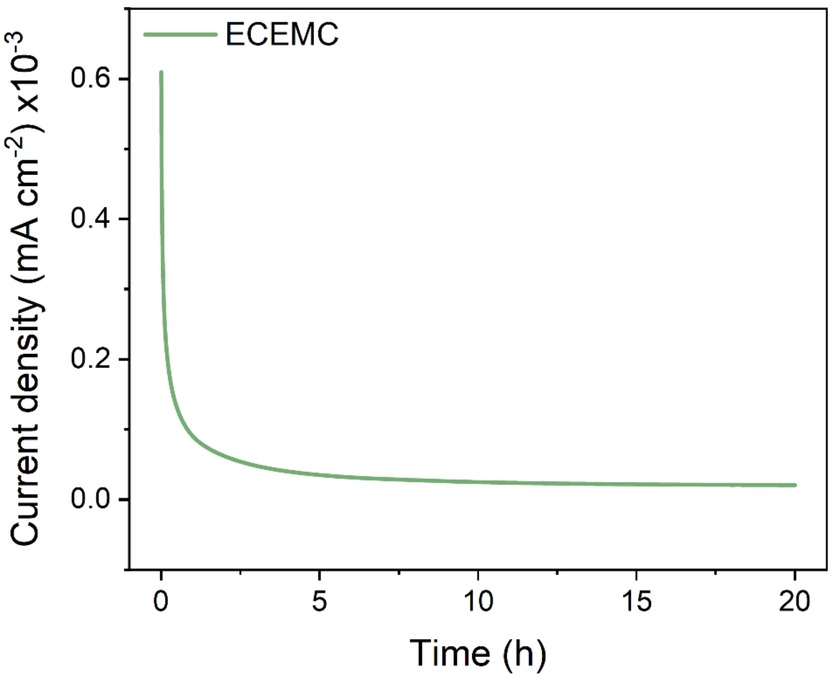

Figure S 3. Electrochemical stability of binary solvent mixture at 4.2V. Chronoamperogram of the base EC:EMC solvent mixture recorded at a voltage of 4.2 V for 20 h.

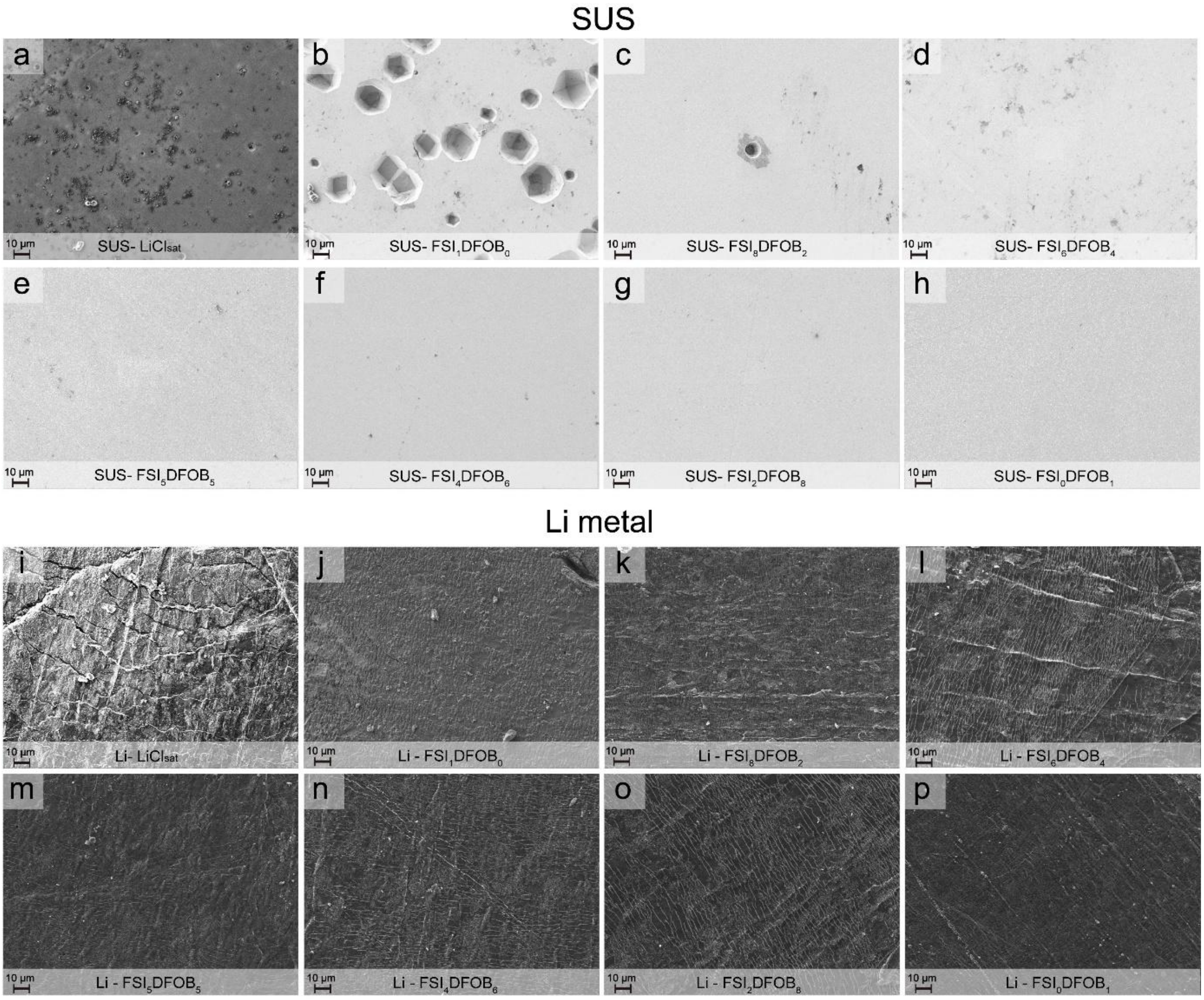

Figure S 4. Morphological characterization of SUS316 spacers and lithium metal anodes after electrochemical test. SEM images of SUS316 spacers recovered from cells containing (a) $LiCl_{sat}$, (b) FSI_1DFOB_0, (c) FSI_8DFOB_2, (d) FSI_6DFOB_4, (e) FSI_5DFOB_5, (f) $FSI_{44}DFOB_6$, (g) FSI_2DFOB_8, (h) FSI_0DFOB_1 after 20 h of CA measurement at 4.2 V and 20 °C. SEM images of Li metals recovered from cells containing (i) $LiCl_{sat}$, (j) FSI_1DFOB_0, (k) FSI_8DFOB_2, (l) FSI_6DFOB_4, (m) FSI_5DFOB_5, (n) $FSI_{44}DFOB_6$, (o) FSI_2DFOB_8, (p) FSI_0DFOB_1 after 20 h of CA measurement at 4.2 V and 20 °C.

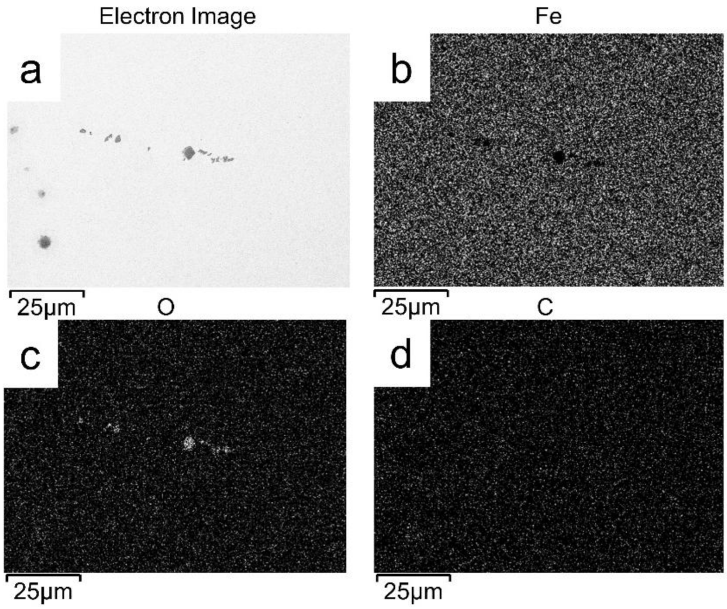

Figure S 5. EDX elemental mapping of the SUS316 surface after electrochemical test. EDX images of the SUS316 spacer harvested from cells after 20 h of CA measurements at 4.2 V and 20 °C with 5 ppm LiCl in the electrolytes. (a) Electron image. (b) EDX image of Fe element. (c) EDX image of O element. (d) EDX image of C element.

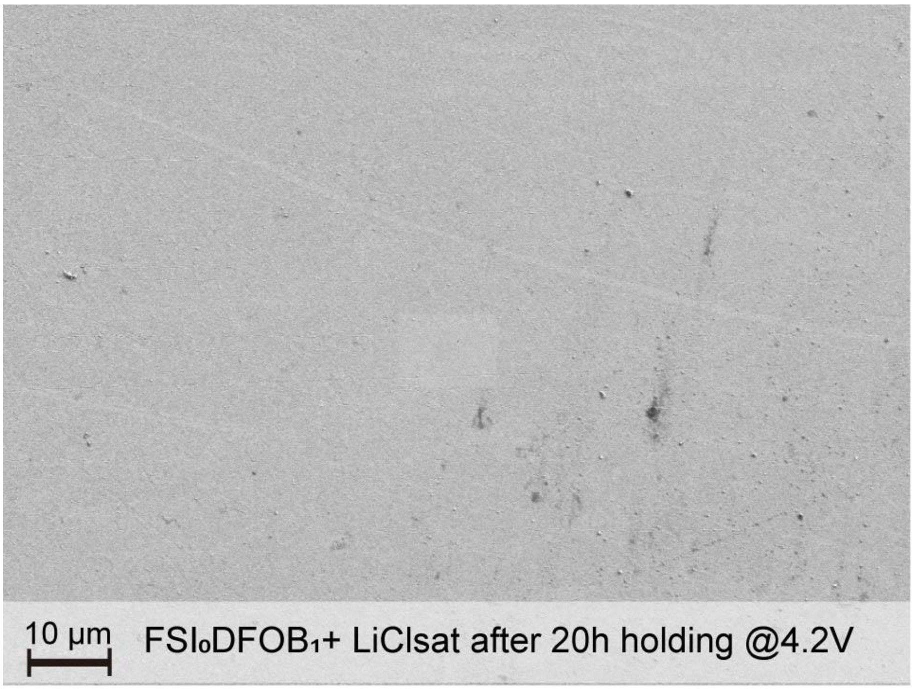

Figure S 6. Morphological characterization of SUS316 spacers after electrochemical test. SEM images of SUS316 spacers recovered from 20 h CA measurement at 4.2 V and 20 °C with FSI_0DFOB_1+ $LiCl_{sat}$ electrolyte.

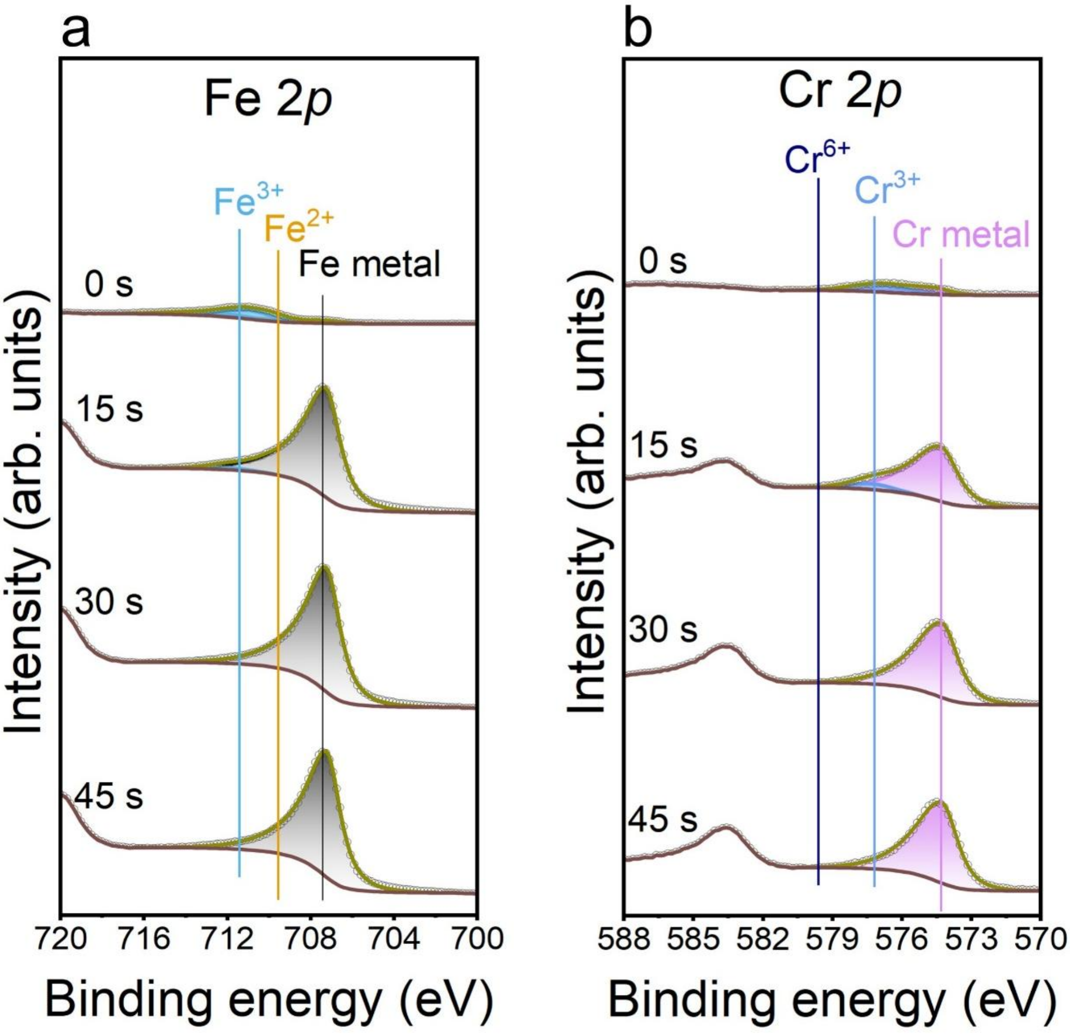

Figure S 7. XPS spectra of pristine SUS316. Selected (a) Fe 2*p* and (b) Cr 2*p* XPS spectra of pristine SUS316 at 0 s, 15 s, 30 s and 45 s etching time.

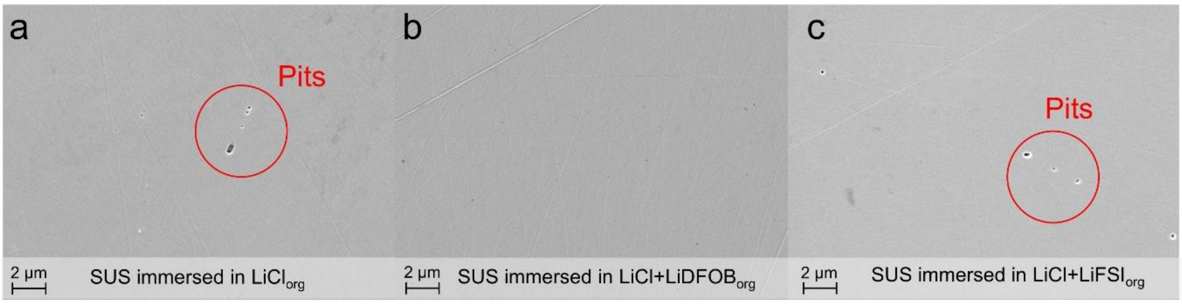

Figure S 8. Morphological characterization of SUS316 spacers after storage test. SEM image of SUS316 surface after 1 month immersed at 25 °C in (a) $LiCl_{org}$ (100 ppm LiCl in EC/EMC 3:7 by wt%), (b) LiCl+$LiDFOB_{org}$ (100 ppm LiCl + 0.5 M LiDFOB in EC/EMC 3:7 by wt%) and (c) LiCl+$LiFSI_{org}$ (100 ppm LiCl + 0.5 M LiFSI in EC/EMC 3:7 by wt%) electrolytes. All samples were rinsed with EMC and dried under vacuum before imaging.

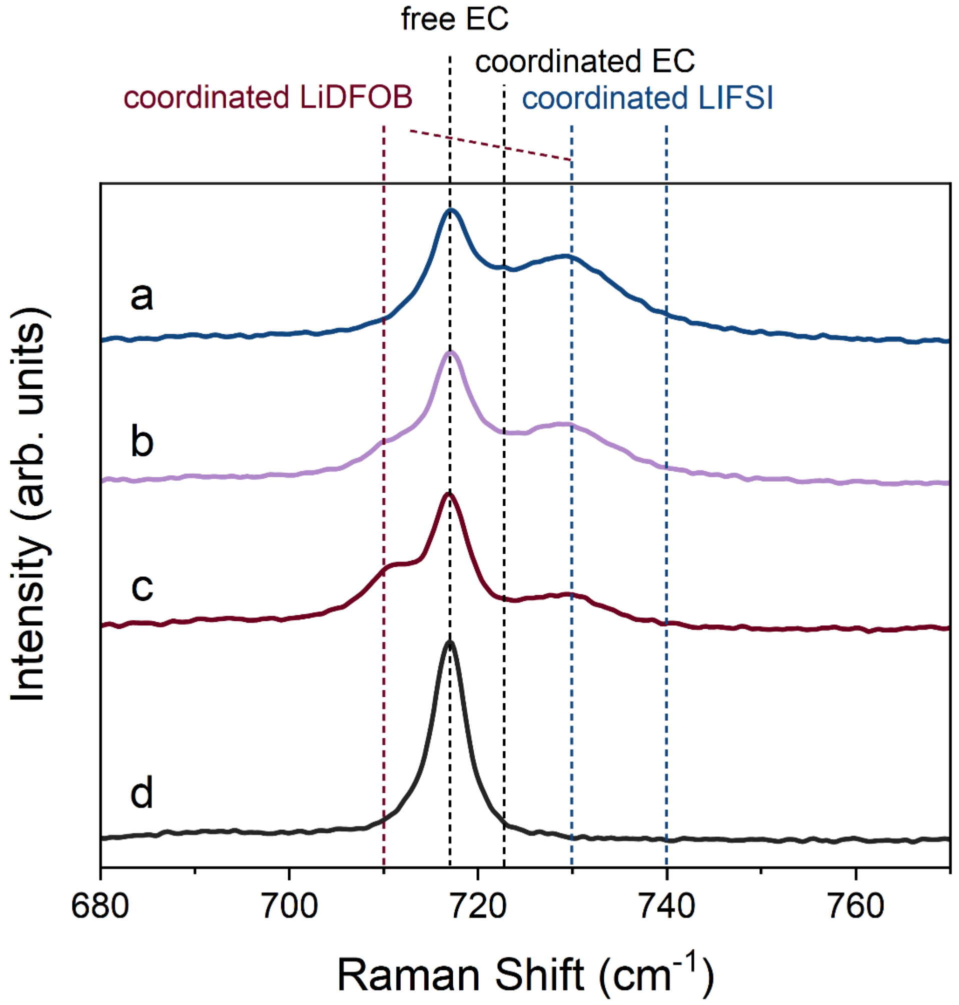

Figure S 9. Raman spectra for fresh electrolytes. Selected Raman spectra of different conducting salt containing electrolytes (a) FSI_1DFOB_0, (b) FSI_5DFOB_5, (c) FSI_0DFOB_1 and (d) 100 ppm LiCl.

The band shown at ≈716 cm^{-1} can be attributed to the C=O ring bending vibration in EC while the band at ≈723 cm^{-1} can be explained by the coordination between free EC and Li^+ ion upon the addition of lithium salts. In electrolytes containing LiDFOB, the bands attributed to the ring breathing of $DFOB^-$ anion are fitted at ≈710 cm-1 and ≈725-734 cm^{-1}, corresponding to the coordinated LiDFOB[1]. The first band of coordinated LiDFOB at ≈710 cm^{-1} can be attributed to the solvent-separated ion pairs (SSIPs, an anion interacting with less than one Li^+ ion) or contact-ion pairs (CIPs, an anion interacting with one Li^+ ion) while another band at ≈725-734 cm^{-1} is attributed to the aggregates (AGGs, an anion interacting with two or more Li^+ ions)[1]. In electrolytes containing LiFSI, additional peaks attributed to coordinated LiFSI are fitted at ≈724-734 and ≈740 cm^{-1}, corresponding to the CIP and AGG, respectively[1]. With this understanding, the solvation structure of electrolytes upon the addition of different lithium salts can be characterized. The spectra of electrolytes containing 100 ppm LiCl have only a band fitted to free EC, suggesting that solvation structures are not enough to be detectable at the ppm level of LiCl (Figure S 9d). The spectra of FSI_1DFOB_0 electrolyte comprise large area peaks of free EC, solvated EC, and CIP while only a small area of AGG from FSI^- anions (Figure S 9a). As for the spectra of FSI_0DFOB_1 electrolyte, a large area of free EC, SSIPs/CIPs and AGGs can be observed, with only a small amount of solvated EC, suggesting the lower dissociation ability of LiDFOB in the organic carbonated solvents compared to LiFSI (Figure S 9c). In the blended salt electrolyte FSI_5DFOB_5, both $DFOB^-$ and FSI^- anions and the EC solvent are involved in the coordination with Li^+ ion, highlighting the contribution of both anions within the solvation structure (Figure S 9b).

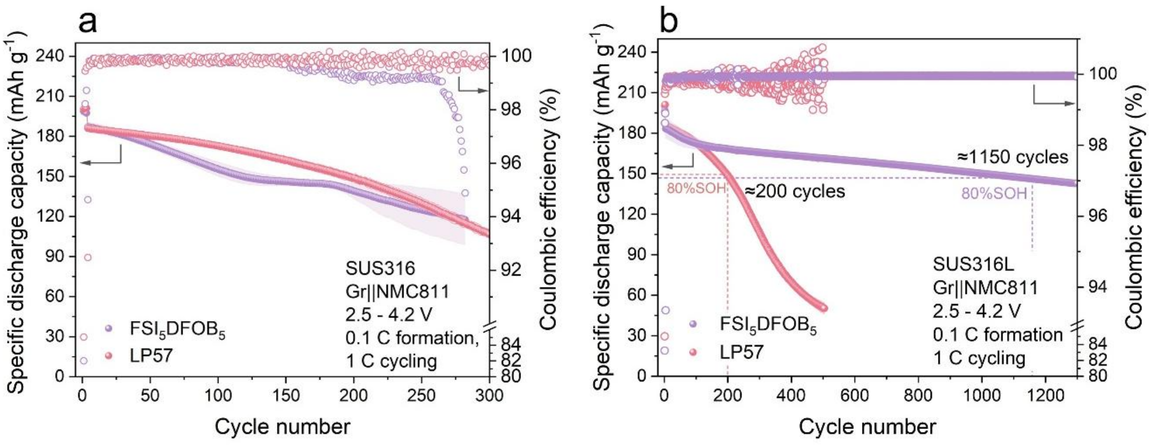

Figure S 10. Validation of LiDFOB-containing electrolytes in Gr||NMC811 coin cells. (a) Specific discharge capacity vs. cycle number curves of coin cells containing SUS316 parts with FSI5DFOB5 and LP57 electrolytes. (b) Specific discharge capacity vs. cycle number curves of Gr||NMC811 coin cells containing SUS316L parts with FSI5DFOB5 and LP57. 1C = 200 mA g^{-1} for NMC811 positive electrode. Abbreviation: SOH: state of health.

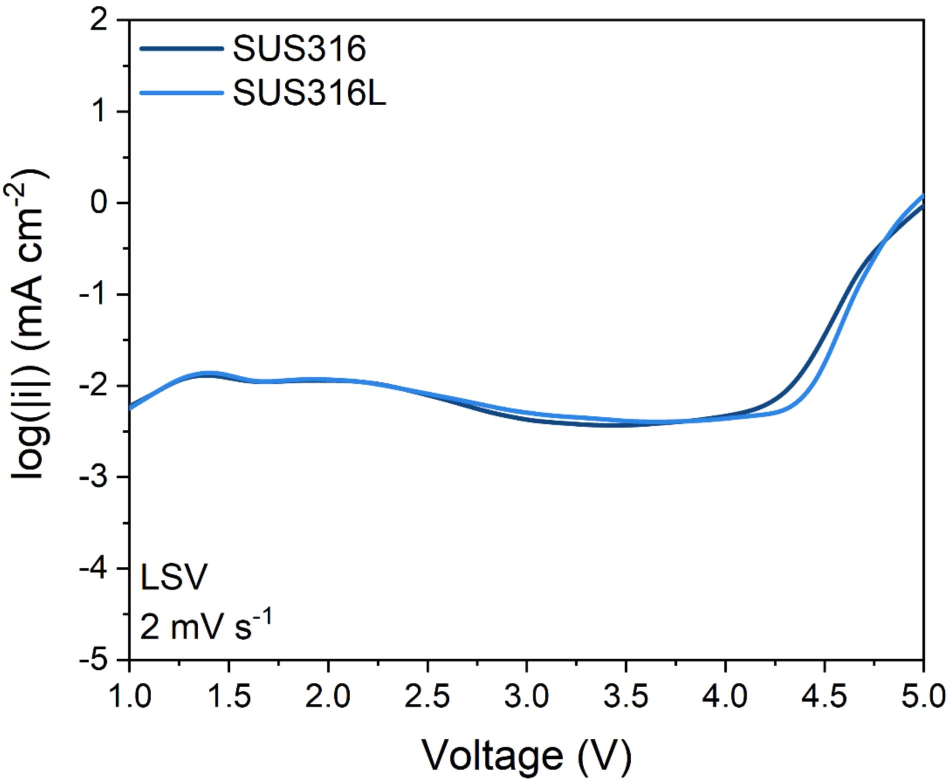

Figure S 11. Electrochemical stability of SUS316 and SUS316L in LiFSI-based electrolytes. Linear sweep voltammogram of cells containing SUS316 and SUS316L as working electrodes in FSI_1DFOB_0 electrolyte.

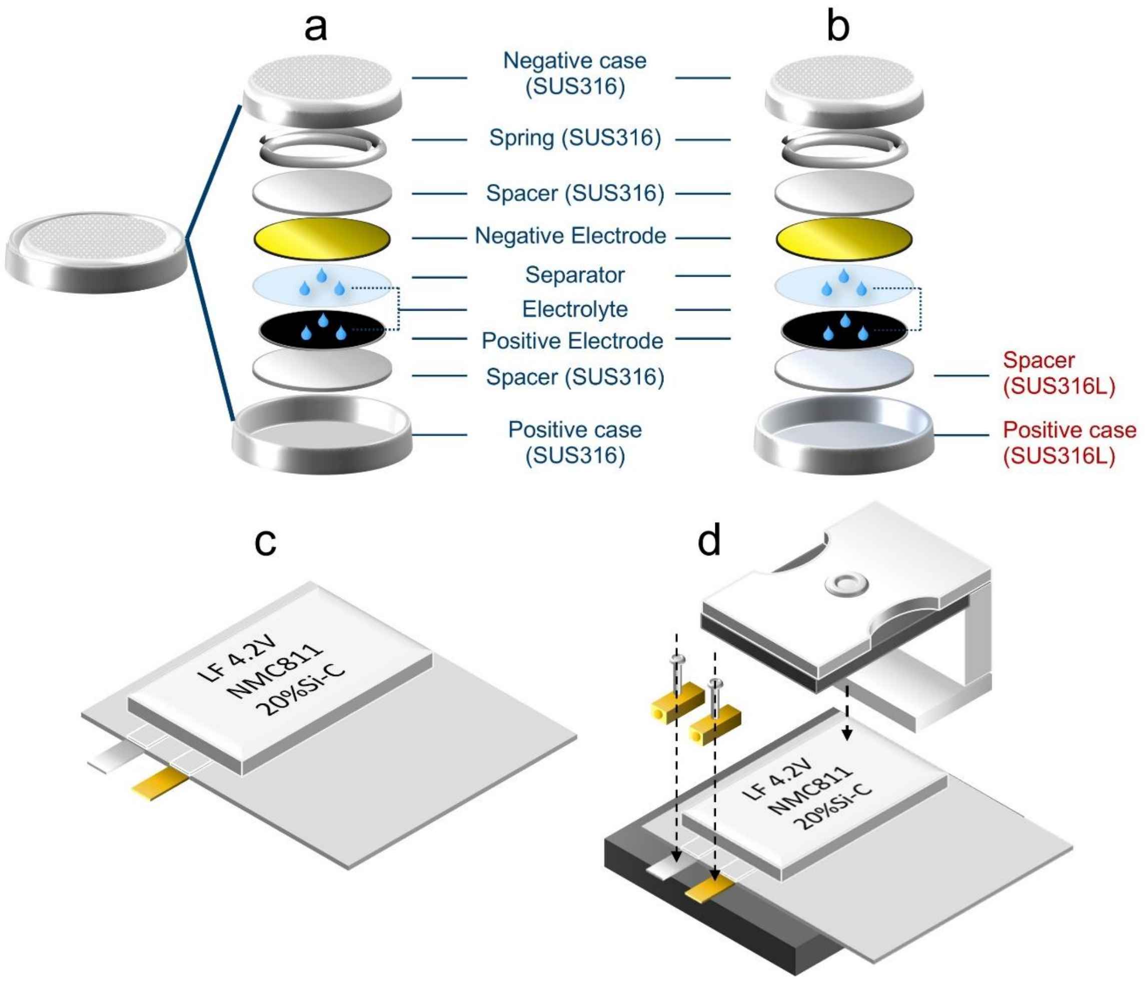

Figure S 12. Schematic illustration of cell configurations and experimental mounting setup. Illustration of the coin cell setup for galvanostatic cycling stability test with (a) SUS316 components and (b) SUS316L components. (c) Illustration of Li-FUN pouch cells. (d) Representation of how the cells are securely mounted to the custom-made cell holder.

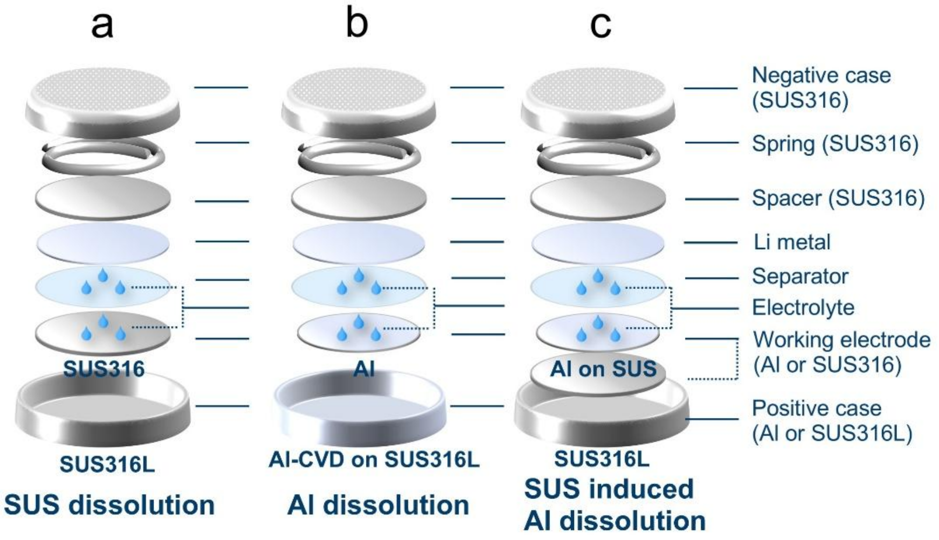

Figure S 13. Schematic illustration of cell configurations for metal dissolution study. Illustration of the coin cell setup for (a) SUS dissolution, (b) Al dissolution and (c) SUS induced Al dissolution studies. Abbreviation: CVD: chemical vapor deposition.

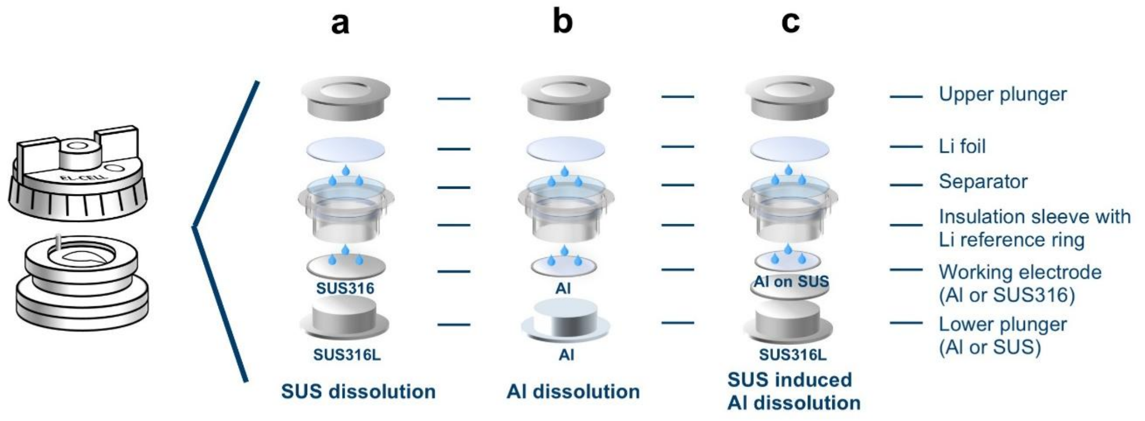

Figure S 14. Schematic illustration of PAT cell configurations for metal dissolution study. Illustration of the PAT cell setup from EL-CELL GmbH for (a) SUS dissolution, (b) Al dissolution and (c) SUS induced Al dissolution studies.

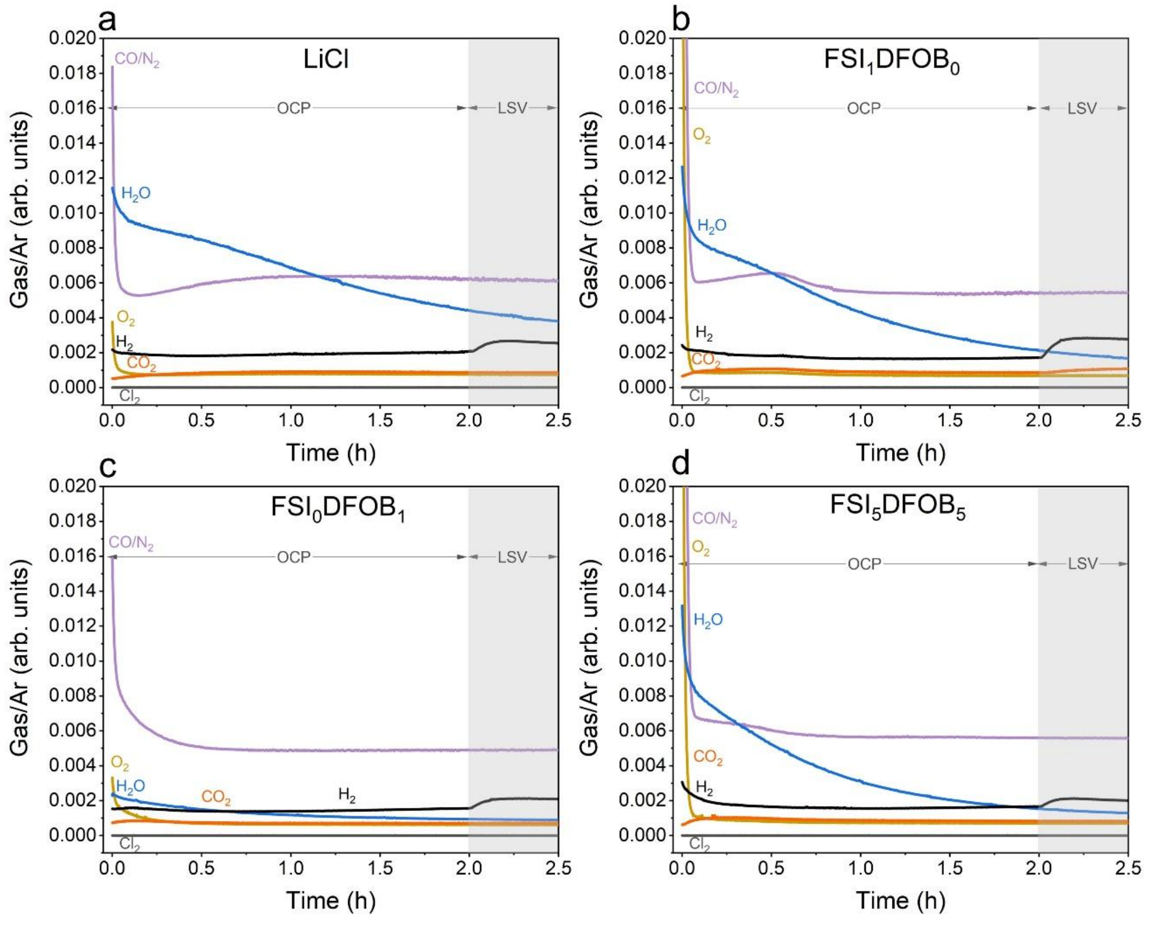

Figure S 15. DEMS analysis of gas evolution. Operando gaseous evolution of the DEMS cell containing (a) LiCl, (b) FSI_1DFOB_0, (c) FSI_0DFOB_1, (d) FSI_5DFOB_5 electrolytes at open circuit potential (OCP) for 2 h, followed by linear sweep voltammetry (LSV). Gas signals are normalized with Ar to eliminate the fluctuation of carrier gas.

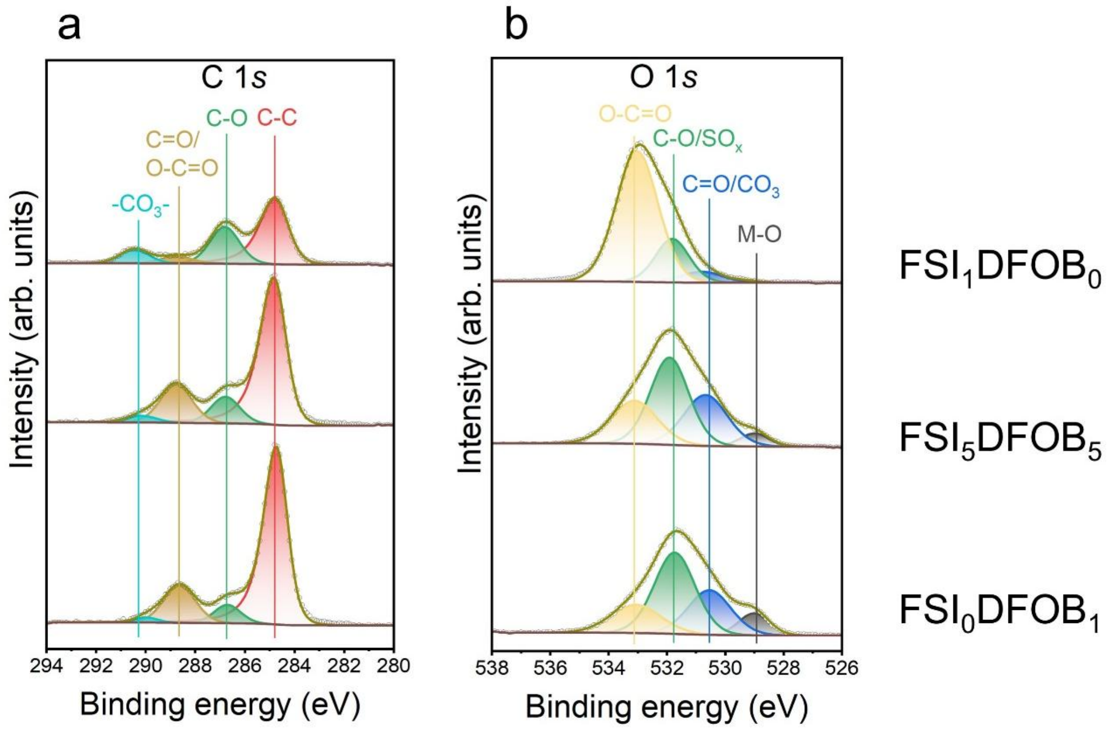

Figure S 16. XPS spectra of harvested SUS316 after electrochemical test. Selected (a) C 1*s* and (b) O 1*s* XPS spectra of the harvested SUS316 spacers from the cells containing FSI_1DFOB_0 (top), FSI_5DFOB_5 (middle) and FSI_0DFOB_1 (bottom) electrolytes after 20 h at 4.2 V and 20 °C.

Supplementary Tables

Table S 1. Elemental distribution weight percentage on the surface of SUS316 collected by EDX.

	C (%)	O (%)	Fe (%)	Cr (%)	Ni (%)	F (%)	S (%)
Pristine	2.55	1.12	68.00	17.04	11.29	0.00	0.00
$LiCl_{sat}$	23.18	32.38	31.00	0.00	5.00	0.00	0.00
FSI_1DFOB_0	3.49	2.82	55.54	14.54	8.83	0.41	3.17
FSI_2DFOB_8	2.71	2.56	67.12	11.46	8.14	1.42	1.09
FSI_5DFOB_5	2.22	1.61	66.86	11.33	7.79	1.48	0.85
FSI_0DFOB_1	1.94	2.44	68.54	10.91	7.92	0.43	0.00

Table S 2. Elemental distribution on the surface of Li collected by EDX.

	Li (%)	C (%)	O (%)	F (%)	N (%)	S (%)	Fe (%)
$LiCl_{sat}$	0.00	38.34	44.65	0.00	0.00	0.00	17.01
FSI_1DFOB_0	0.00	16.70	46.75	4.95	1.51	9.73	20.36
FSI_2DFOB_8	85.76	3.98	7.07	1.71	1.48	0.00	0.00
FSI_5DFOB_5	86.95	3.31	6.22	1.87	1.55	0.00	0.00
FSI_0DFOB_1	80.31	8.86	7.73	1.55	1.25	0.00	0.00

Supplementary Note 1: Investigation of SUS Inhibition Mechanism of LiDFOB

Extended CA measurements conducted for up to 1,000 h with samples containing FSI_6DFOB_4 and FSI_4DFOB_6 electrolytes reveal the important role of LiDFOB in suppressing the SUS dissolution. A slight increase in current is observed after 200 h for FSI_6DFOB_4 and 500 h for FSI_4DFOB_6 (Figure S 17a). The positive correlation between the hours for dissolution starting and LiDFOB amounts reveals that LiDFOB may act as a sacrificial salt additive to protect the SUS against "aggressive anions". Considering the fact that the boron-containing film can be observed on the SUS316 surface with LiDFOB-containing electrolyte, it is assumed that this film can protect the SUS from the attack of aggressive Cl^- and FSI^- anions. To further confirm it, the SUS spacer was pretreated with FSI_0DFOB_1 electrolyte under LSV up to 5 V (referred to pre@SUS) (Figure S 17c). Subsequent XPS analysis of pre@SUS confirmed the presence of a boron-containing surface film, characterized by B-O and B-F, indicative of LiDFOB decomposition (Figure S 17b). However, when the pretreated pre@SUS spacer was reassembled into a cell using the FSI_1DFOB_0 electrolyte, an even higher current density compared to a cell with an untreated spacer during the CA measurements (Figure S 17d) was shown. Such an unexpected behavior indicates that the boron-containing surface film, while also protecting the SUS spacer against the aggressive anions, may also compromise the integrity of the native protective oxides of chromium on the SUS surface, resulting in a less effective surface film that is more prone to the attack of Cl^- and FSI^- anions.

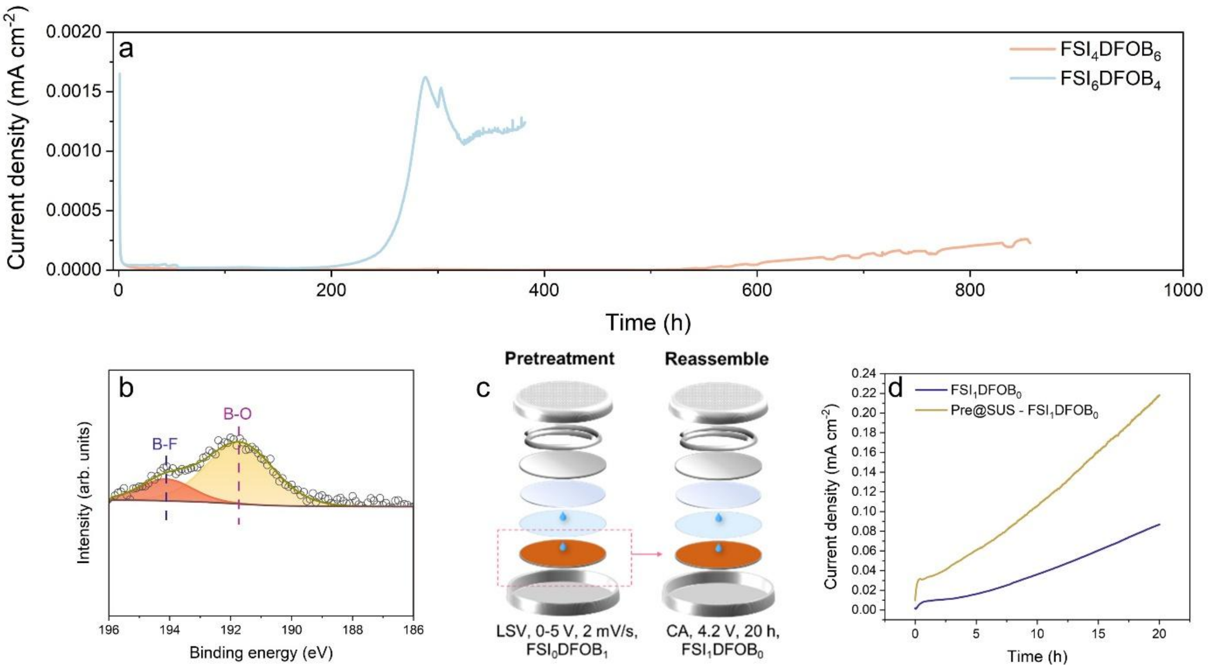

Figure S 17. Investigation of SUS inhibition mechanism of LiDFOB. (a) Chronoamperograms of cells using SUS316 spacer as working electrode with FSI_4DFOB_6 and FSI_6DFOB_4 electrolyte recorded at 4.2 V for 1,000 h. (b) B 1*s* spectra of the SUS316 spacer after pretreatment with FSI_0DFOB_1 electrolyte. (c) Illustration of the pretreatment of SUS316 spacer and reassembling process. The pretreated spacer after linear sweep voltammetry (LSV) is assembled in a new cell for chronoamperometry (CA) with a different electrolyte. (d) Comparison of LSV curves using untreated SUS316 and pre@SUS as working electrodes and FSI_1DFOB_0 electrolyte.

Supplementary Note 2: Investigation of SUS induced Al Dissolution

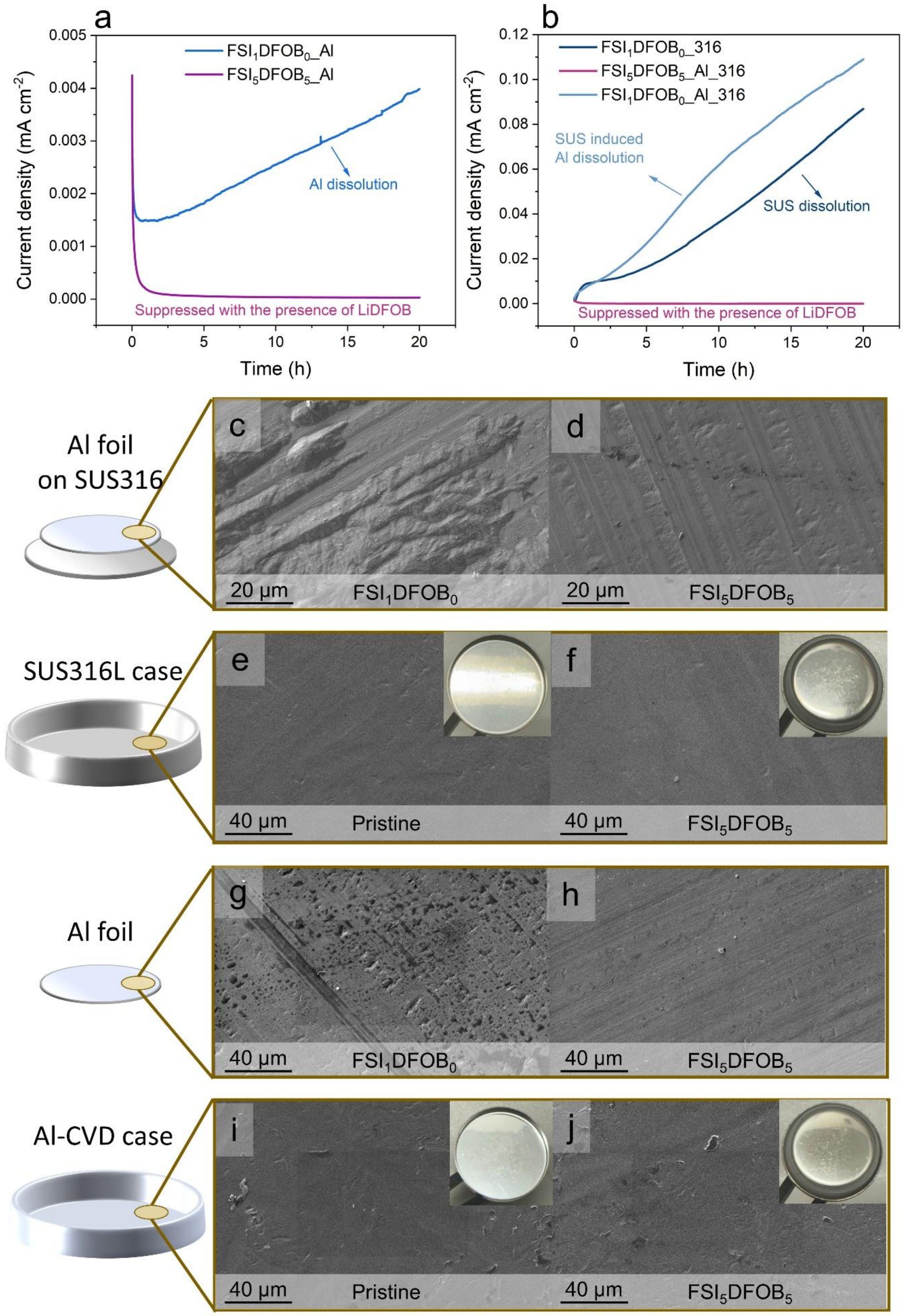

Figure S 18. Investigation of SUS induced Al dissolution. Chronoamperograms of cells with (a) Al foil or (b) Al foil on top of SUS316 space as a working electrode with FSI_1DFOB_0 and FSI_5DFOB_5 electrolyte recorded at 4.2 V.

SEM image of Al foil recovered from (c) FSI_1DFOB_0_Al_316 and (d) FSI_5DFOB_5_Al_316 cells after 20 h CA measurements at 4.2 V and 20 °C. SEM image of the inner surface of the SUS316L case from (e) pristine sample and (f) FSI_5DFOB_5_Al_316 cell after 20 h CA measurements at 4.2 V and 20 °C. SEM image of Al foil recovered from (g) FSI_1DFOB_0_Al and (h) FSI_5DFOB_5_Al cells after 20 h CA measurements at 4.2 V and 20 °C. SEM image of the inner surface of the Al-CVD case from (i) pristine sample and (j) FSI_5DFOB_5_Al cell after 20 h CA measurements at 4.2 V and 20 °C. Abbreviation: CVD: chemical vapor deposition.

Modified coin cell setups using Al foil as the working electrode were used for these experiments, as shown in Figure S 13b, c. The variations of current density during the CA measurements are displayed for the FSI_1DFOB_0 and FSI_5DFOB_5 electrolytes, with and without SUS spacers (Figure S 18a,b). In cells using Al foil solely, the addition of LiDFOB notably inhibits the Al dissolution caused by the presence of LiFSI, as confirmed by the suppressed current density during CA measurements (Figure S 18a) and intact Al foil (Figure S 18h) as well as Al-CVD case (Figure S 18j) harvested after CA measurements. This is consistent with findings reported in the literature[2–4].

Notably, when Al foil is placed on top of SUS316 spacer in coin cells, the cells containing FSI_1DFOB_0 electrolyte display considerably higher dissolution behavior compared to Al, as evidenced by the 30 times higher current density after 20 h (≈0.11 mAh cm^{-2} for FSI_1DFOB_0_Al_316 *vs.* ≈0.004 mAh cm^{-2} for FSI_1DFOB_0_Al) (Figure S 18b). This effect can be attributed to the formation of galvanic cells between Al and SUS, which lowers the overpotential for the oxidation of Al and consequently enhances the dissolution of Al at high voltage[5]. SEM images further support these findings, showing a rough and irregular surface (Figure S 18c), differs from the pits observed from the Al foil recovered from sample FSI1DFOB0_Al (Figure S 18g). This special morphology is similar to the stress-induced fracture observed on the uncoated side of positive electrode recovered from cells assembled with SUS316 and containing FSI_1DFOB_0 (Figure S 23h). Similarly, when LiDFOB is included in the electrolyte (FSI_5DFOB_5), the coin cell containing Al foil on top of SUS316 spacer shows a very stable current density till the end of measurements, resulting in a smooth Al surface and intact SUS316 case as observed from the SEM images (Figure S 18d,f). This indicates that the inhibiting effects of LiDFOB on SUS dissolution also extends to suppressing Al dissolution in the presence of SUS spacers and coin cell cases.

Supplementary Note 3: Investigation of the Impact of Solvent Decomposition on Metal Dissolution

During the LSV measurements, a pronounced current increase is observed at ≈3.5 V in the cell containing $LiCl_{sat}$ electrolyte (Figure 1a). This rise in current indicates the destabilization of native oxide layer on SUS and the onset of SUS dissolution. Such a current increase can be triggered either by solvent decomposition or by the presence of Cl^- anion. Proton released form the solvent oxidation can initiate Al dissolution at a similar voltage onset[6], which could be one of the reasons for SUS dissolution observed here. To validate this hypothesis, CA and CV measurements were conducted using fluorinated carbonate solvents, which possess higher oxidative stability and lower tendency for proton release[7]. In these experiments, EC and EMC in the FSI_1DFOB_0 electrolyte were replaced by their fluorinated analogues, i.e. FEC and FEMC, in the same ratio (denoted as $F\_FSI_1DFOB_0$).

First, the Al foil was used as working electrode to confirm prior literature evidence that proton release contributes to Al dissolution. For this purpose, a cell equipped with an Al-CVD case, an Al spacer, and an Al working electrode was assembled (Figure S 1b). As shown in Figure S 19a, a lower current density is observed for cells containing $F\_FSI_1DFOB_0$ during the CA measurements with Al foil as the working electrode, suggesting that the Al dissolution is reduced in the presence of fluorinated solvents. However, the oxidative current in cells containing $F\_FSI_1DFOB_0$ increases continuously over time, indicating that while the dissolution rate is reduced but still not completely mitigated. This suggests that proton release is important but not the only factor governing Al dissolution. Further CV measurements revealed the voltage onsets of Al dissolution and also the passivation behaviors. Figure S 19b,c showed the cyclic voltammograms of cells containing FSI_1DFOB_0 and $F\_FSI_1DFOB_0$. The shape of the first cycle differs from the subsequent ones and the anodic current is observed starting from 3 V *vs*. Li/Li^+. Such an early onset has been attributed to the oxidation of organic carbonate-based solvents or the solvent impurity species (e.g. EG or MeOH), which can generate protons and destabilize the native Al_2O_3 layer on the surface of the Al current collector[6–8]. When EC/EMC mixture is replaced by FEC/FEMC (Figure S 19e), the onset of anodic current shifted ~0.1 V to higher voltage, indicating that fluorinated solvents with their higher oxidative stability indeed suppress proton release and delay Al_2O_3 layer breakdown. Interestingly, both LiFSI-based electrolytes showed a decrease in current during the reverse scan (4.3 V to 2.5 V *vs*. Li/Li^+), and the current is further decreased in the subsequent scans. This trend resembles the behavior observed in cells containing FSI_5DFOB_5, with a slightly higher current density maximum, indicating that the Al foil is gradually passivated during the cycles (Figure S 19d). This behavior contrasts with the observation of Scheer *et al.* and Nyholm *et al*., where increased current during reverse scan and accelerated Al dissolution were reported[6,8]. This different trend observed here is most likely linked to the low Cl^- anion impurity (7.49 ppm detected by IC measurement) in the LiFSI used in our study. Han *et al.* demonstrated that the extra pure LiFSI (0.45 ppm Cl^-) does not dissolve Al during CV scans between 3 – 5 V *vs*. Li/Li^+, while Al dissolution is observed when 50 ppm Cl^- is added into electrolytes[9]. Although the LiFSI used in our study is not as pure as in the publication of Han *et al*., its low Cl^- content is sufficient to slow down the Al dissolution upon consecutive scans. The occurrence of Al dissolution was evidenced for the cells with FSI_1DFOB_0 by the clear pits and deposits observed on the Al foil after three CV scans (Figure S 19g), in contrast with pits-free surface observed on Al foils collected from cells with LiDFOB containing electrolyte (FSI_5DFOB_5) (Figure S 19i). Notably, less pits were found on Al foils in cells with $F\_FSI_1DFOB_0$ (Figure S 19h), consistent with the reduced proton generation and delayed Al dissolution in fluorinated solvents.

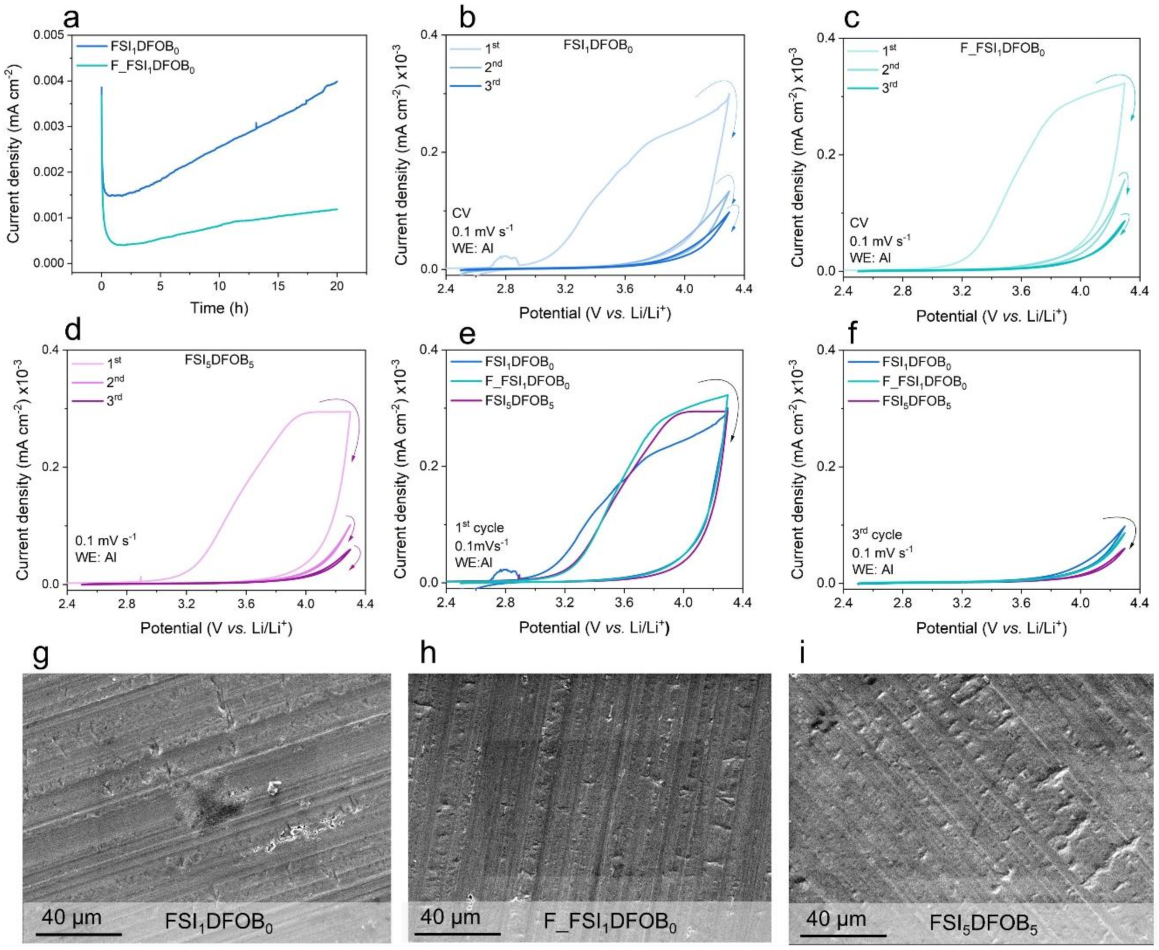

Figure S 19. Investigation of the Impact of solvent decomposition on Al dissolution. (a) Chronoamperograms of cells with Al foil as a working electrode (WE) with FSI_1DFOB_0 and $F\_FSI_1DFOB_0$ electrolyte recorded at 4.2 V. Cyclic voltammetry (CV) curves of cells containing Al as working electrodes with (b) FSI_1DFOB_0 and (c) $F\_FSI_1DFOB_0$ and (d) FSI_5DFOB_5 electrolyte. Comparison of the (e) first cycle and (f) third cycle of cyclic voltammetry for cells with considered electrolytes. SEM image of Al foil recovered from (g) FSI_1DFOB_0 (h) $F\_FSI_1DFOB_0$ and (i) FSI_5DFOB_5 after three CV cycles (scan range of 2.5 to 4.3 V *vs.* Li/Li^+, cells disassembled at 2.5 V *vs.* Li/Li^+).

After confirming the positive effect of fluorinated solvents in mitigating the Al dissolution, we then examined whether suppressing proton release would also affect the SUS dissolution. In strong contrast to the behavior observed for Al, the cells containing $F\_FSI_1DFOB_0$ exhibited a substantially higher current density than those with non-fluorinated FSI_1DFOB_0 during CA measurements (Figure S 20a). This clearly indicates that increasing the oxidative stability of the solvent and thereby reducing proton release does not mitigate SUS dissolution. Instead, the dissolution is even accelerated in the fluorinated solvent system. Additional CV measurements further support this conclusion. For both LiFSI-based electrolytes, recorded CV curves displayed the typical dissolution behavior, characterized with a pronounced increase in current during the reverse scan, forming a hysteresis loop (Figure S 20b,c). While cells with LiDFOB containing electrolyte FSI_5DFOB_5 showed suppressed current density across CV scan range (Figure S 20d). Notably, in the first cycle, cells with $F\_FSI_1DFOB_0$ show an earlier voltage onset for SUS dissolution and reached a much higher anodic current maximum compared to the cells with FSI_1DFOB_0 (Figure S 20e). When the scan is reversed, cells with $F\_FSI_1DFOB_0$ still exhibit relatively large currents over a wide potential window and show a pronounced hysteresis between forward and reverse scans, characteristic of a strongly activated dissolution process. Subsequent scans further confirmed a higher anodic current maximum for cells with $F\_FSI_1DFOB_0$ (Figure S 20f). These electrochemical results are consistent with the post-mortem surface analysis. SEM images of SUS316 after CV cycles provided direct

evidence of pitting in the presence of both LiFSI-based electrolytes (Figure S 20g,h), whereas a clean and pits-free surface is observed on SUS spacer when LiDFOB is present (Figure S 20i). However, the sample using F_FSI$_1$DFOB$_0$ exhibited larger and more densely distributed pits, indicating a more severe dissolution process (Figure S 20h). These results suggested that the dissolution mechanism in SUS is different from Al and is governed by the FSI- (and to some extend Cl^- impurities), rather than by proton release associated with solvent oxidation.

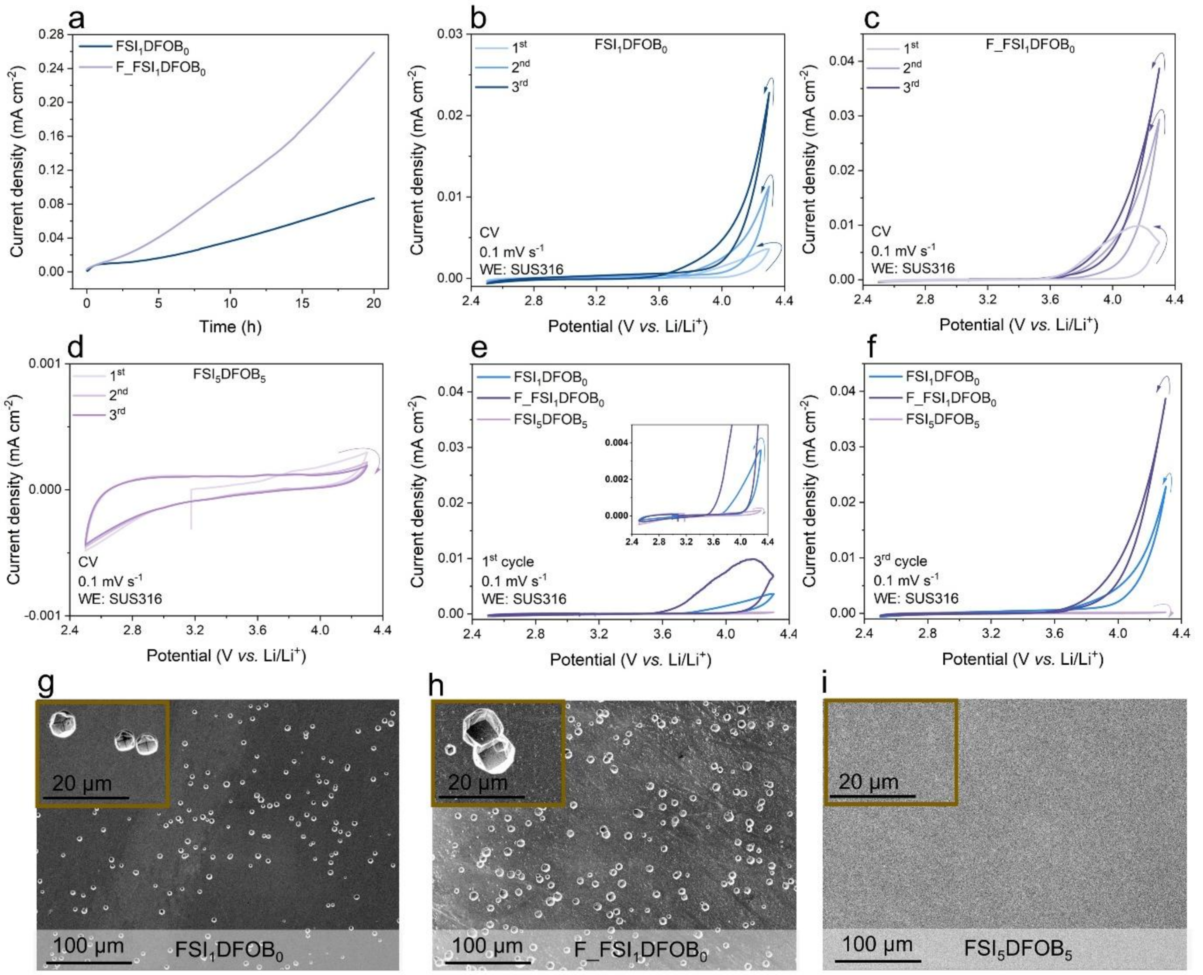

Figure S 20. Investigation of the Impact of solvent decomposition on SUS dissolution. (a) Chronoamperograms of cells with SUS316 spacer as a working electrode (WE) with FSI$_1$DFOB$_0$ and F_FSI$_1$DFOB$_0$ electrolyte recorded at 4.2 V. Cyclic voltammetry (CV) curves of cells containing SUS316 spacer as working electrodes with (b) FSI$_1$DFOB$_0$ and (c) F_FSI$_1$DFOB$_0$ and (d) FSI$_5$DFOB$_5$ electrolyte. Comparison of the (e) first cycle and (f) third cycle of cyclic voltammetry for cells with considered electrolytes. SEM image of SUS316 spacer recovered from (g) FSI$_1$DFOB$_0$ (h) F_FSI$_1$DFOB$_0$ and (i) FSI$_5$DFOB$_5$ after three CV cycles (scan range of 2.5 to 4.3 V *vs.* Li/Li$^+$, cells disassembled at 2.5 V *vs.* Li/Li$^+$).

At this stage, we can conclude that fluorinated solvent slightly mitigates Al dissolution but significantly accelerate SUS dissolution, owing to the fundamentally different dissolution mechanism of Al and SUS in LiFSI-based electrolyte. It is therefore worth to evaluate the complex dissolution behavior when both Al and SUS are presented. Figure S 21a shows the CA measurements using Al on top of SUS as working electrode. Although the F_FSI$_1$DFOB$_0$ can mitigate the Al dissolution, as shown in Figure S 20a, it accelerates the dissolution process when both Al and SUS are presented. Additional CV measurements show a similar trend compared to the cells with sole SUS as working electrode (Figure S 20b-f). Post-mortem SEM after CV scans reveals larger deposits on Al and larger pits on SUS for cells with F_FSI$_1$DFOB$_0$ compared to non-fluorinated samples (Figure S 20g,h,j,k). These observations confirm that SUS dissolution has a more severe problem and can dominate the dissolution in the cell. As a

result, improving solvent oxidative stability slightly mitigates Al dissolution but has little effect, or even has detrimental effect on SUS stability when fluorinated solvents are employed.

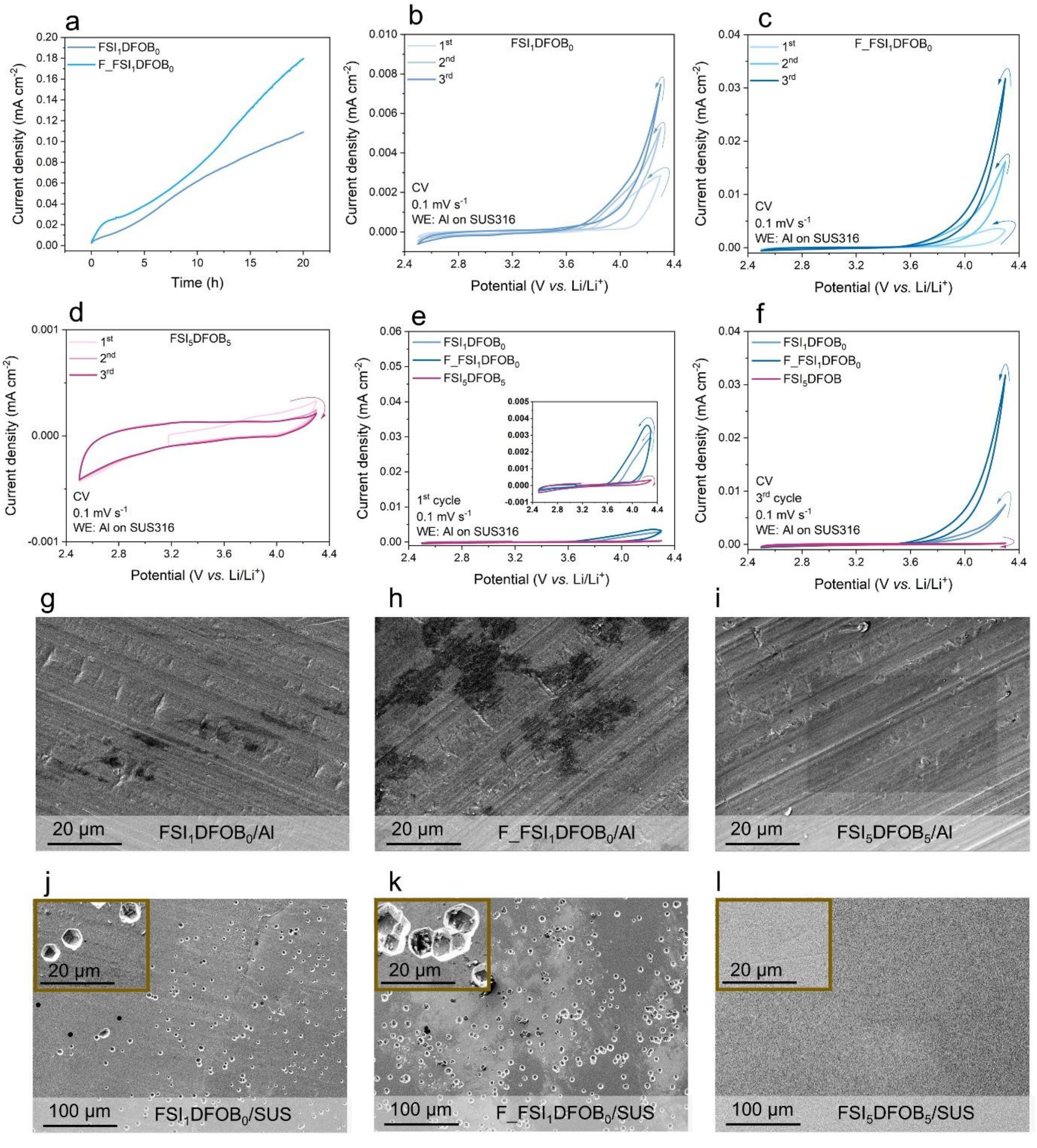

Figure S 21. Investigation of the Impact of solvent decomposition on SUS induced Al dissolution. (a) Chronoamperograms of cells with Al foil on top of SUS316 space as a working electrode (WE) with FSI_1DFOB_0 and $F\_FSI_1DFOB_0$ electrolyte recorded at 4.2 V. Cyclic voltammetry (CV) curves of cells containing Al foil on top of SUS316 space as working electrodes with (b) FSI_1DFOB_0 and (c) $F\_FSI_1DFOB_0$ and (d) FSI_5DFOB_5 electrolyte. Comparison of the (e) first cycle and (f) third cycle in the cyclic voltammetry measurement for cells with considered electrolytes. SEM image of Al foil recovered from (g) FSI_1DFOB_0 (h) $F\_FSI_1DFOB_0$ and (i) FSI_5DFOB_5 after three CV cycles (scan range of 2.5 to 4.3 V *vs.* Li/Li$^+$). SEM image of SUS316 spacer recovered from (j) FSI_1DFOB_0 (k) $F\_FSI_1DFOB_0$ and (l) FSI_5DFOB_5 after three CV cycles (scan range of 2.5 to 4.3 V *vs.* Li/Li$^+$). All cells for SEM imaging were disassembled at 2.5 V *vs.* Li/Li$^+$.

Supplementary Note 4: Investigation of Al Dissolution during the Cycling Process

It has been noticed that Gr||NMC811 cells containing FSI_1DFOB_0 are not able to charge to 4.2V, and a pronounced rise in anodic current is observed during the CV step (Figure 7a). This anodic current may be introduced by SUS or Al dissolution. To distinguish the contribution from SUS and Al dissolution, cells containing FSI_1DFOB_0 electrolytes after the charging process were disassembled and analyzed both the SUS spacer and coated Al foil using SEM Given that Al dissolution may also occur on the coated side of Al and potentially trigger the binding-force failure between the positive electrode materials and the binder, the active material was carefully removed using tweezers to expose the underlying Al foil. This sample preparation enabled direct observation of the Al surface morphology on the coated side. Pristine NMC811 served as the reference, and two cells assembled with different coin cell grades (SUS316 vs. SUS316L) were disassembled to compare the dissolution behavior of SUS and aluminum in the presence of different SUS grades. The cells assembled with SUS316L parts exhibited an increase in anodic current at the 3rd charging step, suggesting higher dissolution resistance compared to cells using SUS316 parts (Figure S 22).

Inspection of the Al foil from the NMC811 coated side revealed visible surface scratches and residual active materials remaining after mechanical removal of the positive electrode layer in the pristine NMC811 sample (Figure S 23a). Additionally, small indentations were identified on the Al surface, likely resulting from the compression of active material particles into the Al foil during the calendaring process. In sample FSI_1DFOB_0/316, pronounced Al dissolution was observed, characterized by the formation of large crevices on the coated side (Figure S 23b). Notably, similar crevices were also observed on the uncoated side of Al foil (Figure S 23h). The morphology of these crevices presented localized breakdown due to stress-induced fracture, which is a common characteristic of pitting corrosion[10,11]. In contrast, no obvious pitting or crevices formation was observed on both sides of Al foil from sample FSI_1DFOB_0/316L (Figure S 23c,i).

Upon examining the SUS spacers from both cells, extensive pitting was observed, predominantly distributed along the edges of the spacer (Figure S 23n,o). This is consistent with expectations, as the central area of the SUS spacer is covered by the positive electrode, while the edge areas remain direct exposure to the electrolytes. The morphology and size of the pits were similar between sample FSI_1DFOB_0/316 and FSI_1DFOB_0/316L, but considerable deposits were observed on the SUS spacer for sample FSI_1DFOB_0/316. In general, these results indicate that the pronounced rise in anodic current observed in Figure 7a (using SUS316 as coin cell parts) originated from both SUS dissolution and Al dissolution, which contributed to cell failure in the presence of LiFSI-based electrolytes. Interestingly, the use of SUS316L (higher dissolution resistance) appears to prevent the Al dissolution. Nevertheless, SUS dissolution is evident on both cells, suggesting that the SUS dissolution is more severe compared to the Al dissolution and is the dominant factor responsible for the rise of anodic current in Figure 7a.

To further validate that the SUS dissolution is the primary cause of cell failure and the observed rise in anodic current, we introduced $LiPF_6$ as a co-salt into the LiFSI-based electrolyte to passivate the Al current collector. $LiPF_6$ is widely recognized as an effective Al dissolution inhibitor and is usually used as an additive or co-salt to protect the Al current collector in LiFSI and LiTFSI-based electrolytes[8,12,13]. To ensure the comparability, we used an equivalent salt ratio in the blended electrolyte (0.5M LiFSI + 0.5M $LiPF_6$ in EC:EMC 3:7 by wt%) and noted as FSI_5PF_5. As shown in Figure S 22, the voltage vs. time profiles of cells containing FSI_5PF_5 still exhibit a pronounced rise in anodic current during the constant voltage step. This indicates that the presence of $LiPF_6$ could not prevent cell failure during the charging step. Furthermore, SEM images confirmed that no evidence of Al dissolution was observed on the current collector from positive electrode (Figure S 23d,j), while clear pitting was still observed on the SUS spacer (Figure S 23p). These results reinforce the conclusion that SUS dissolution plays the dominant role in cell failure in LiFSI-based electrolytes.

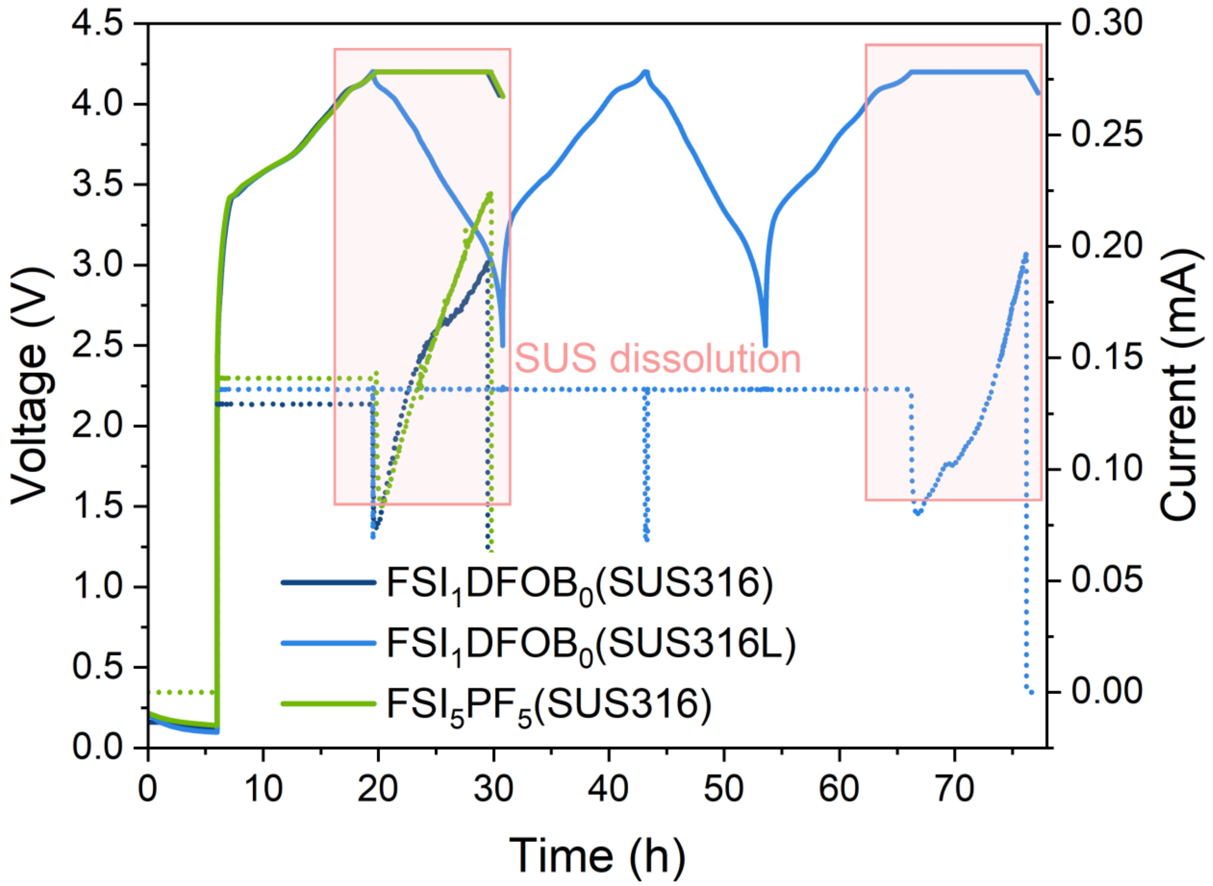

Figure S 22. Voltage *vs.* time profiles of Gr||NMC811 cells with SUS316/316L parts using FSI_1DFOB_0 and FSI_5PF_5 electrolytes. 1C = 200 mA g^{-1} for NMC811 positive electrode.

Similarly, a rapid drop in Coulombic efficiency (CE) in cells with FSI_5DFOB_5 and SUS316 con cell parts may also be introduced by Al dissolution, as shown in Figure S 10a. To confirm the reason, cells containing FSI_5DFOB_5 after the cycling procedure were disassembled and the dissolution conditions of SUS and Al foil were examined.

Two cells using different con cell parts (SUS316 and SUS316L) were disassembled. No clear evidence of Al dissolution was observed on both sides of the electrodes in both cases (Figure S 23e,f,k,l). With respect to the SUS spacers, no distinct pitting was identified on either SUS316 or SUS316L (Figure S 23q,r). However, prominent blackish deposits were detected along the edge of the SUS316 spacer, indicative of iron oxidation. Furthermore, the surface of the SUS316 spacer appeared more uneven compared to the pristine sample in Figure S 28a, indicating that the dissolution may have occurred in the form of crevice corrosion rather than pitting. Notably, no obvious current increase was observed during cycling, implying that the SUS dissolution in cells with FSI_5DFOB_5 proceeded much more slowly compared to that in cells with FSI_1DFOB_0. This is likely to explain the absence of pits and the presence of only surface deposits. Based on observations from SEM, it can be concluded that the SUS dissolution is the main reason for the rapid drop in Coulombic efficiency (CE) in Figure S 10a.

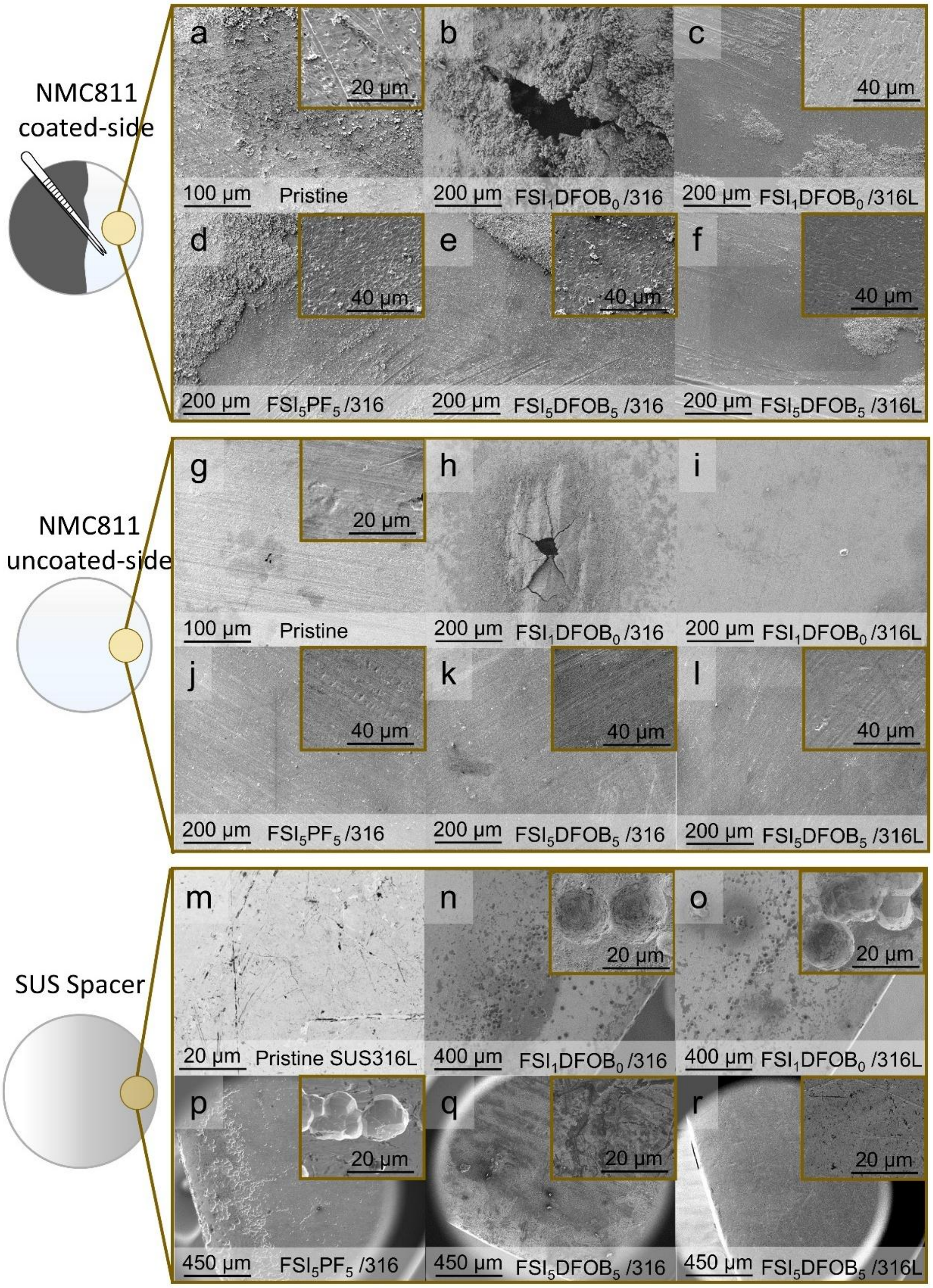

Figure S 23. Morphological characterization of Al foil from NMC811 positive electrode and SUS316 spacers after different cycle numbers. SEM image of Al foil from the (a) coated side and (b) uncoated side of the pristine NMC811 electrode, and (m) pristine SUS316L spacer. The following images correspond to the Al foil from the coated side, uncoated side, and the SUS spacer after disassembling different cells: (b, h, n) cells with FSI_1DFOB_0 and SUS316 coin cell parts (disassembled after 1st cycle); (c, I, o) cells with FSI_1DFOB_0 and SUS316L coin cell parts (disassembled after 3rd cycle); (d, j, p) cells with FSI_5PF_5 and SUS316 coin cell parts (disassembled after 1st cycle); (e, k, q) cells with FSI_5DFOB_5 and SUS316 coin cell parts (disassembled after 282th cycle); (f, l, r) cells with FSI_5DFOB_5 and SUS316L coin cell parts (disassembled after 1365th cycle). All cells were disassembled in the discharged state.

Supplementary Note 5: Investigation of the Anti-Dissolution Performance of LiDFOB in Half cells.

The roll-over effect in LIBs involves multiple issues, including the loss of active materials from both positive electrode and negative electrode, loss of lithium inventory, corrosion of current collectors, electrolyte decomposition. To eliminate the influence of graphite electrode, the anti-dissolution performance of LiDFOB was evaluated in Li||NMC811 cells. Interestingly, stable cycling at 4.2 V was achieved in cells containing the FSI_1DFOB_0 electrolyte when lithium metal was used as the counter electrode (Figure S 24a), which is different compared to cells Gr||NMC811 cycled at 4.2V (Figure 7a). With a three-electrode cell revealed that the actual potential of NMC811 vs. Li reference electrode is 4.28 V, rather than the nominal 4.2V, due to the higher potential of lithiated graphite (Figure S 24d). To further confirm it, the upper cutoff voltage (UCV) was increased to 4.3 V, which again resulted in a pronounced rise in anodic current (Figure S 24e). Although the Li||NMC811 cells could sustain cycling at 4.2V, the low CE (<98%) observed in both SUS316 and SUS316L parts suggests the existence of SUS/Al dissolution. The cell using SUS316 parts ends at the 32th cycle with a rise of anodic current during the constant voltage step (Figure S 24b). In comparison, cells with SUS316L parts demonstrate improved CE and extend the cycling stability up to 133 cycles. However, the rise of anodic current at the 38th cycle also points to the existence of SUS/Al dissolution (Figure S 24c).

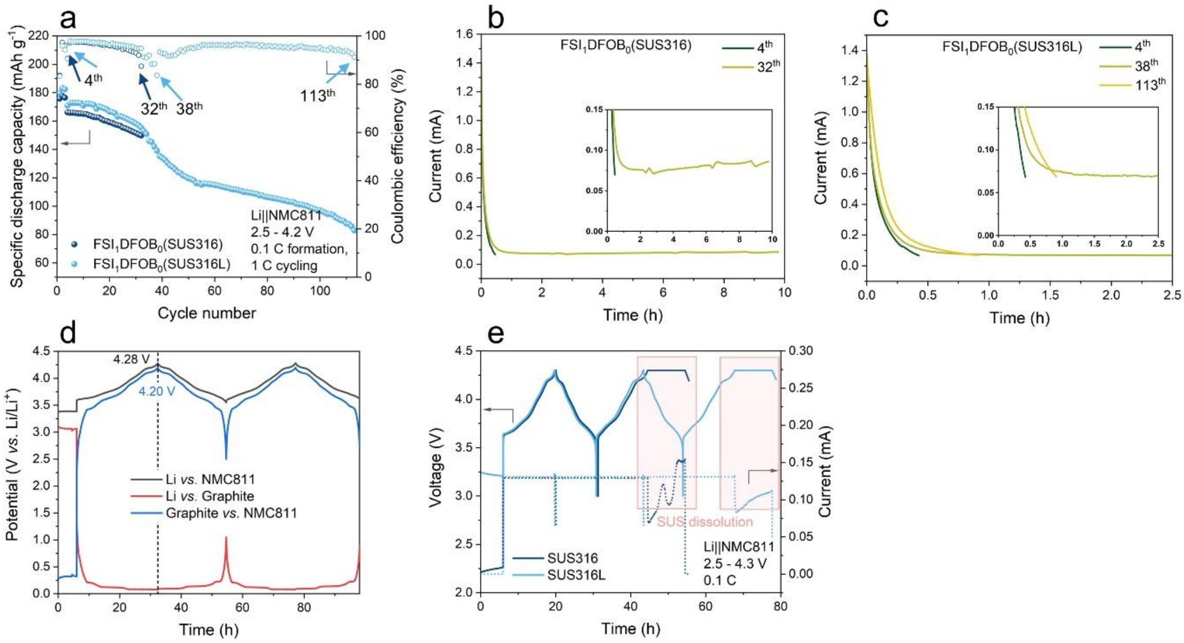

Figure S 24. Comparative cycling performance of Li||NMC811 cells with SUS316/316L parts. (a) Specific discharge capacity vs. cycle number for Li||NMC811 cells containing SUS316/316L parts with FSI_1DFOB_0 electrolyte. Current vs. time profile during the constant voltage step at various cycles for cells assembled with (b) SUS316 and (c) SUS316L con cell parts. (d) Voltage vs. time profile of a three-electrode cell using LP57 electrolyte. (e) Voltage *vs.* time profiles of Li||NMC811 cells with SUS316/316L parts using FSI_1DFOB_0 electrolytes. 1C = 200 mA g^{-1} for NMC811 positive electrode.

As for cells with FSI_5DFOB_5, a similar trend could also be observed compared to the Gr‖NMC811 in Figure S 10a. Li‖NMC811 cells SUS316L coin cell parts demonstrate extended cycling stability compared to cells assembled with SUS316 coin cell parts (Figure S 25a). In particular, Li‖NMC811 cells assembled with SUS316 parts have a fast drop of CE at ≈100 cycles, consistent with the behavior observed in Gr‖NMC811 cells. In addition, Li‖NMC811 cells assembled with SUS316L parts with FSI_5DFOB_5 electrolytes show improved performance compared to those with LP57, indicating that the optimized electrolyte is also effective in

Li‖NMC811 cell configuration. Notably, the use of different SUS grades has no impact on the galvanostatic cycling performance of cells containing LP57, consistent with observations from full cells. However, the overall life time of the Li||NMC811 cells (no matter which electrolyte) is inferior to the Gr||NMC811 cell. The faster fading observed in the Li||NMC811 cells is attributed to the lithium dendrite growth, which can lead to possible micro-shorts. Furthermore, the formation of high surface area lithium (HSAL) accelerates electrolyte decomposition[14]. It should be noted that 1C charge current rate was employed in the half cell cycling stability measurements, which is critical for the Li metal negative electrode[15]. Zhang *et al.* show that slow charging (0.2C) and fast discharging (3C) considerably improve the performance of Li||NMC811 cells, while fast charging (1C) and slow discharging (0.33C) deteriorate the galvanostatic cycling performance due to the formation of HSAL[15].

A Similar rapid capacity fading was also observed in Li||Gr cells. As shown in Figure S 25b, none of the tested cells maintained a stable cycle beyond 100 cycles, with performance degradation even more severe than that observed in Li||NMC811 cells. Smith *et al.* observed similar trends by comparing the long term cycling performance between Li||NMC811, Gr||NMC811 and Li||Gr cells and conclude this as the higher areal capacity of graphite, thicker surface layer on graphite side which leads to more side reactions on the Li||Gr cells[14]. Despite the overall instability of this configuration, differences in performance were observed among cells employing different electrolytes. Cells with LP57 have a capacity decay already after 40 cycles, while cells with FSI_1DFOB_0 and FSI_5DFOB_5 show a good cycling stability until the roll-over due to the dendrite growth under the applied 1C current rate.

To further confirm the roll-over failure in Li||Gr cells, the cycled cells were disassembled and both graphite and Li metal were analyzed by SEM imaging. It is in general that no clear changes are observed on the graphite particles compared to the pristine graphite, except that the cells with FSI_5DFOB_5 show particle fracture (Figure S 26). Furthermore, The Li metal surface in the cells with FSI_5DFOB_5 shows clear cracking and presence of HSAL (Figure S 26h), These features are known to accelerate electrolyte decomposition and promote electrode degradation. EDX further confirmed the presence of substantial electrolyte decomposition products on both the graphite and lithium metal surfaces (Table S 3 and Table S 4). Based on these observations, it can be concluded that the roll-over failure is primarily caused by the formation of HSAL on the lithium metal surface. Overall, the FSI_5DFOB_5 electrolyte demonstrates favorable compatibility with graphite negative electrode materials.

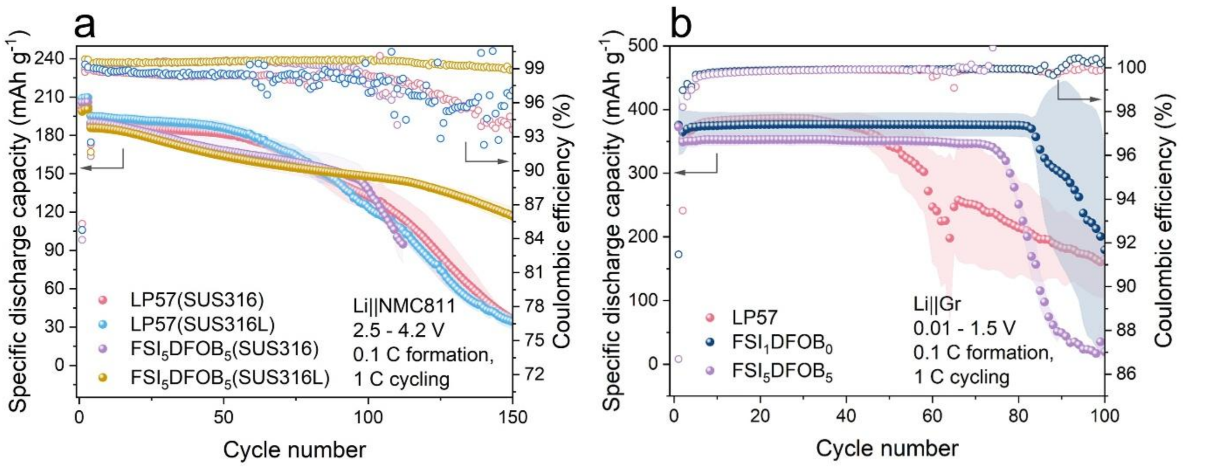

Figure S 25. Investigation of the SUS inhibitive effect of LiDFOB containing electrolytes in half cells. (a) Specific discharge capacity *vs.* cycle number curves of Li||NMC811 cells containing SUS316/316L parts with FSI_5DFOB_5 and LP57 electrolytes. (b) Specific discharge capacity *vs.* cycle number curves of Li||Gr cells containing SUS316 parts with FSI_1DFOB_0, FSI_5DFOB_5 and LP57 electrolytes. 1C = 200 mA g^{-1} for NMC811 positive electrode. 1C = 350 mA g^{-1} for graphite electrode.

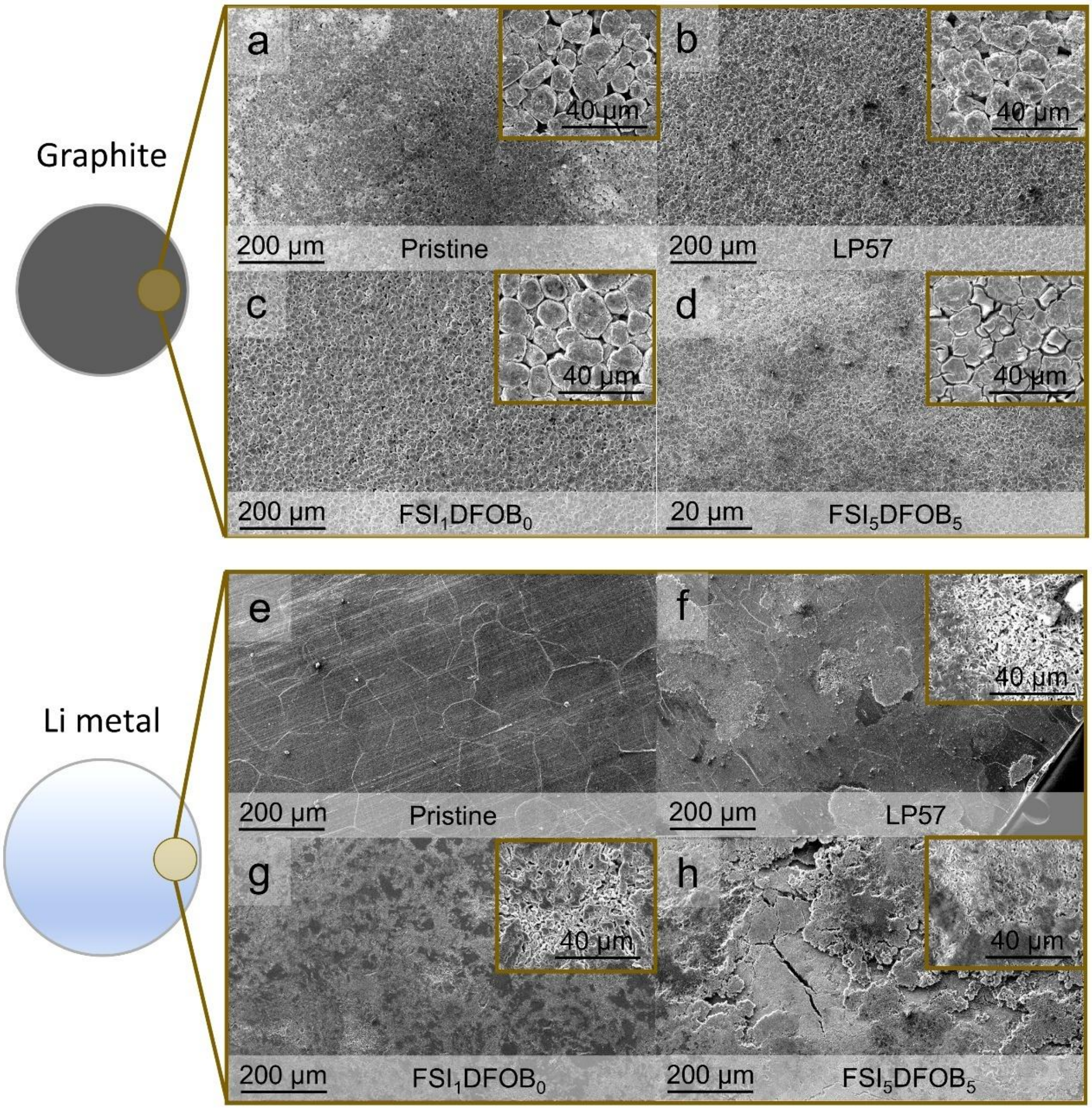

Figure S 26. Morphological characterization of graphite and Li metal after long-term cycling in Li||Gr cells. SEM image of (a) pristine graphite and (e) pristine Li metal. The following images correspond to the graphite and Li metal recovered from different cells at end of life (EOL) at discharged state: (b,f) cells with LP57; (c,g) cells with FSI_1DFOB_0; (d,h) cells with FSI_5DFOB_5.

Table S 3. Elemental distribution weight percentage on the surface of Gr collected by EDX.

	C (%)	O (%)	F (%)	N (%)	S (%)	B (%)	P (%)
LP57	57.21	25.65	12.53	0	0	0	4.6
FSI_1DFOB_0	60.36	15.52	13.01	1.30	9.81	0	0
FSI_5DFOB_5	34.50	30.70	12.50	1.50	6.00	14.8	0

Table S 4. Elemental distribution on the surface of Li collected by EDX.

	C (%)	O (%)	F (%)	N (%)	S (%)	B(%)	P (%)
LP57	16.50	39.20	32.90	0	0	0	11.40
FSI_1DFOB_0	9.10	51.00	16.90	3.30	19.70	0	0
FSI_5DFOB_5	12.09	65.63	9.19	2.45	6.19	4.4	0

Supplementary Note 6: Impact of Coin Cell Parts on Galvanostatic Cycling Performance

The use of SUS316L in coin cell components has a negligible impact on electrochemical performance for cells using the LP57 (1M $LiPF_6$ in EC:EMC, 3:7) electrolyte compared to those with SUS316 parts. Both Gr||NMC811 and Si-C||NMC811 cell chemistries exhibit similar capacity fading trends, indicating that the capacity fading observed in cells containing LP57 electrolyte is unrelated to SUS dissolution.

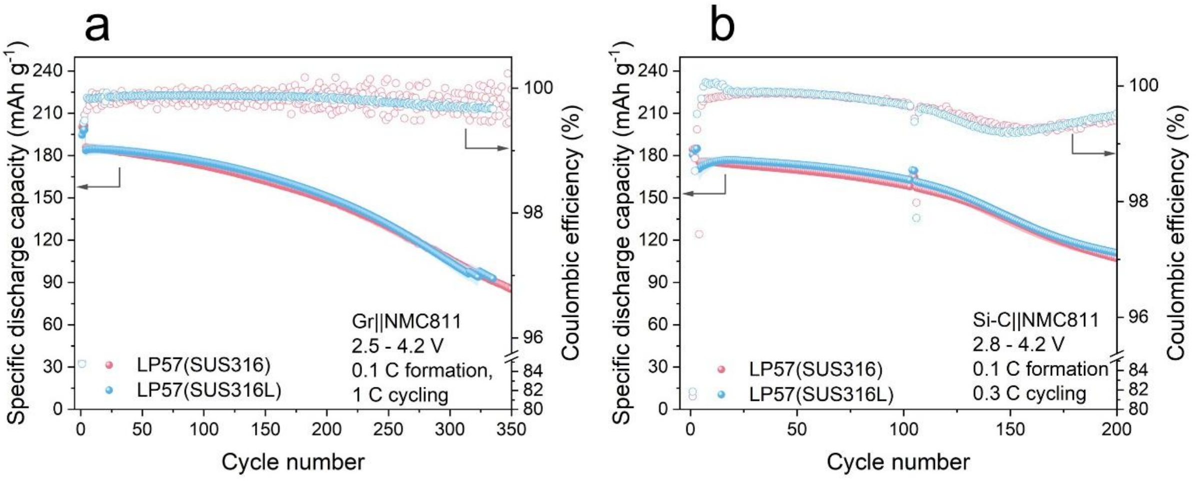

Figure S 27. Impact of SUS grade on galvanostatic cycling performance. Specific discharge capacity *vs.* cycle number of cells containing SUS316L parts with LP57 electrolyte in a) Gr||NMC811 and b) Si-C||NMC811 cell chemistries. 1C = 200 mA g^{-1} for NMC811 positive electrode.

Supplementary Note 7: Investigation of the Impact of Polishing on Reproducibility

SUS316 and SUS316L spacers were polished to reach the mirror quality of the SUS surface, as confirmed by the SEM in Figure S 28. The impact of the surface roughness can be investigated by means of LSV measurements, as shown in Figure S 29. Compared to unpolished samples, the linear sweep voltammogram with polished samples shows improved reproducibility.

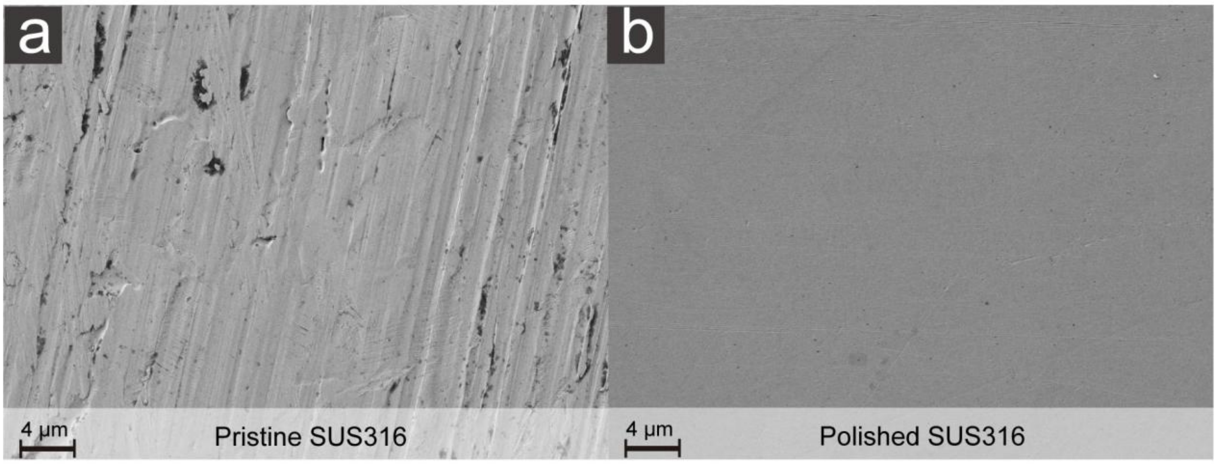

Figure S 28. Morphological characterization pristine SUS316 spacer. SEM images of a) pristine and b) polished SUS316 spacers.

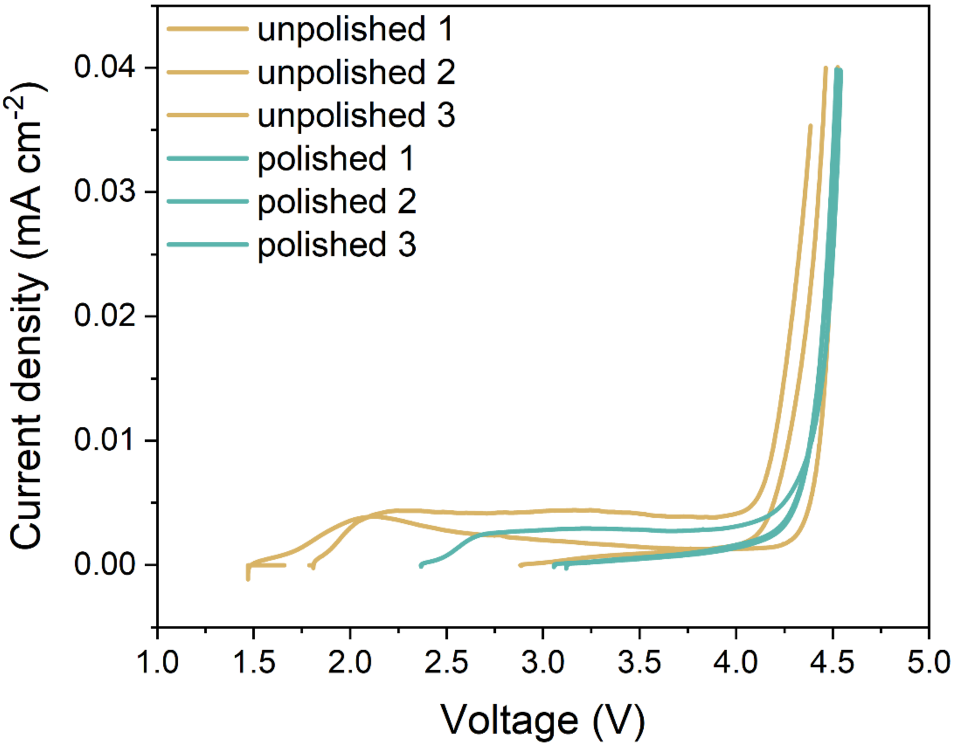

Figure S 29. Impact of SUS surface polishing on electrochemical reproducibility. Linear sweep voltammograms of cells containing pristine and polished SUS316 spacers with FSI_1DFOB_0 electrolyte.

Supplementary Note 8: Investigation of the Impact of Trace Moisture on SUS Dissolution

Karl Fischer titration experiments were conducted for electrolyte components to determine the moisture level of the LiFSI-based electrolyte used in this study. The measured values summarized in Table S 5 indicated that all lithium salts have a very low moisture level and are under the detection limits of the Karl-Fischer titration device. The EC/EMC mixture also showed a quite low moisture level of 16.52 ppm. To ensure that no excess moisture was introduced during the electrolyte preparation process, additional Karl Fischer titration analysis was performed on the formulated electrolytes LP57, FSI_1DFOB_0 and FSI_5DFOB_5. It can be observed that the formulated electrolytes generally maintain low moisture levels, except for FSI5DFOB5, which shows a slightly higher value of 39.32 ppm.

Table S 5. Values from Karl Fischer titration experiments for electrolyte components.

**Components**	**Moisture level (ppm)**
LiFSI	under the detection limits (<10 ppm)
$LiPF_6$	under the detection limits (<10 ppm)
LiDFOB	under the detection limits (<10 ppm)
EC/EMC mixture	16.65
FSI_1DFOB_0	15.12
FSI_5DFOB_5	39.32
LP57	12.35

To investigate the potential impact of trace amounts moisture on SUS dissolution behavior, tris(trimethylsilyl)phosphate (TMSPa) was used as moisture scavenger to further reduce the moisture level in selected electrolytes. TMSPa is known to react with residual water and form trimethylsilanol (TMSOH) and H_3PO_4[16]. Based on the recommendation from the literature, 5 vol % of TMSPa was added on top of electrolytes and their moisture level was examined further by Karl Fischer titration analysis. The results summarized in Table S 6 confirm a reduction in moisture level in both electrolytes after the addition of the TMSPa additive.

Table S 6. Values from Karl Fischer titration experiments for electrolytes with TMSPa additives.

**Components**	**Moisture level (ppm)**
FSI_1DFOB_0 + TMSPa	10.26
FSI_5DFOB_5 + TMSPa	12.34

Further linear sweep voltammetry (LSV) and chronoamperometry (CA) measurements were conducted for the electrolytes containing TSMPa to evaluate the potential impact of trace moisture on SUS dissolution. As shown in Figure S 30, cells with TMSPa-containing electrolytes demonstrated similar electrochemical behavior in both LSV and CA measurements when compared to those without TMSPa. This indicates that the residual moisture content in investigated electrolytes is sufficiently low and does not impact the SUS dissolution behavior under the conditions investigated in this study.

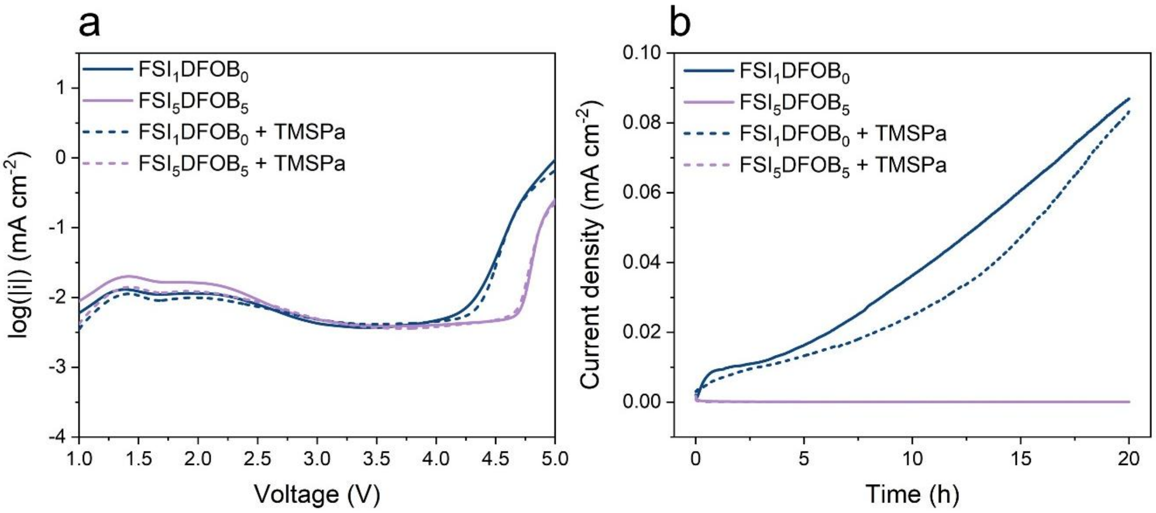

Figure S 30. Impact of trace moisture on SUS dissolution. (a) Linear sweep voltammograms of cells containing SUS316 as working electrode and TMSPa containing electrolytes and (b) corresponding chronoamperograms recorded at 4.2 V for 20 h.

Supplementary References

[1] Z. Chang, X. Li, F. Yun, Z. Shao, Z. Wu, J. Wang, S. Lu, "Effect of Dual-Salt Concentrated Electrolytes on the Electrochemical Performance of Silicon Nanoparticles" *ChemElectroChem* **2020**, *7*, 1135–1141.

[2] K. Park, S. Yu, C. Lee, H. Lee, "Comparative study on lithium borates as corrosion inhibitors of aluminum current collector in lithium bis(fluorosulfonyl)imide electrolytes" *J. Power Sources* **2015**, *296*, 197–203.

[3] S. Shui Zhang, "An unique lithium salt for the improved electrolyte of Li-ion battery" *Electrochem. Commun.* **2006**, *8*, 1423–1428.

[4] X. Liu, C. Shen, N. Gao, Q. Hou, F. Song, X. Tian, Y. He, J. Huang, Z. Fang, K. Xie, "Concentrated electrolytes based on dual salts of LiFSI and LiODFB for lithium-metal battery" *Electrochimica Acta* **2018**, *289*, 422–427.

[5] S. S. Zhang, "A Study on Corrosion of Al-Clad Coin Cell Cases in High-Voltage Li-Ion Battery" *J. Electrochem. Soc.* **2023**, *170*, 110527.

[6] L. Nyholm, T. Ericson, A. S. Etman, "Revisiting the stability of aluminum current collectors in carbonate electrolytes for high-voltage Li-ion batteries" *Chem. Eng. Sci.* **2023**, *282*, 119346.

[7] T. Ma, G.-L. Xu, Y. Li, L. Wang, X. He, J. Zheng, J. Liu, M. H. Engelhard, P. Zapol, L. A. Curtiss, J. Jorne, K. Amine, Z. Chen, "Revisiting the Corrosion of the Aluminum Current Collector in Lithium-Ion Batteries" *J. Phys. Chem. Lett.* **2017**, *8*, 1072–1077.

[8] K. M. Scheer, M. Tulloch, I. Hamam, J. J. Abraham, M. B. Johnson, M. Metzger, "Anodic Dissolution of the Aluminum Current Collector in Lithium-ion Cells with LiFSI, $LiPF_6$, and $LiBF_4$" *J. Electrochem. Soc.* **2025**, *172*, 010511.

[9] H.-B. Han, S.-S. Zhou, D.-J. Zhang, S.-W. Feng, L.-F. Li, K. Liu, W.-F. Feng, J. Nie, H. Li, X.-J. Huang, "Lithium bis(fluorosulfonyl)imide (LiFSI) as conducting salt for nonaqueous liquid electrolytes for lithium-ion batteries: Physicochemical and electrochemical properties" *J. Power Sources* **2011**, *196*, 3623–3632.

[10] P. Marcus, V. Maurice, H.-H. Strehblow, "Localized corrosion (pitting): A model of passivity breakdown including the role of the oxide layer nanostructure" *Corros. Sci.* **2008**, *50*, 2698–2704.

[11] E. Yoon, J. Lee, S. Byun, D. Kim, T. Yoon, "Passivation Failure of Al Current Collector in $LiPF_6$ -Based Electrolytes for Lithium-Ion Batteries" *Adv. Funct. Mater.* **2022**, *32*, 2200026.

[12] C. Behling, J. Lüchtefeld, K. J. J. Mayrhofer, B. B. Berkes, "Quantitative evaluation of LiPF6 as corrosion inhibiting additive for Al in LiTFSI-based battery electrolytes – An on-line mass spectrometric study" *Electrochem. Commun.* **2024**, *159*, 107646.

[13] M. Dahbi, F. Ghamouss, F. Tran-Van, D. Lemordant, M. Anouti, "Comparative study of EC/DMC LiTFSI and LiPF6 electrolytes for electrochemical storage" *J. Power Sources* **2011**, *196*, 9743–9750.

[14] A. Smith, P. Stüble, L. Leuthner, A. Hofmann, F. Jeschull, L. Mereacre, "Potential and limitations of research battery cell types for electrochemical data acquisition" *Batter. Supercaps* **2023**, *6*, DOI 10.1002/batt.202300080.

[15] Y. Zhang, W. Bao, E. Jeffs, B. Liu, B. Han, W. Mai, X. Li, W. Li, Y. Xu, B. Bhamwala, A. Liu, L. Ah, K. Ryu, Y. S. Meng, H. Gan, "Unveiling the impacts of charge/discharge rate on the cycling performance of Li-metal batteries" *ACS Energy Lett.* **2025**, *10*, 872–880.

[16] N. Gogoi, W. Wahyudi, J. Mindemark, G. Hernández, P. Broqvist, E. J. Berg, "Reactivity of organosilicon additives with water in Li-ion batteries" *J. Phys. Chem. C* **2024**, *128*, 1654–1662.
